# The Renaissance of Reproductive Science: Leonardo da Vinci’s Anatomical Contributions

**DOI:** 10.1007/s43032-024-01772-9

**Published:** 2025-01-16

**Authors:** Michael Carroll

**Affiliations:** Department of Life Sciences, Faculty of Science and Engineering, Dalton Building, Manchester Metropolitan University, Chester Street, Manchester, M1 5GD England

**Keywords:** Anatomy, Reproductive science, Renaissance, Art, Leonardo da Vinci

## Abstract

Leonardo de Vinci is considered one of the greatest artists that has ever lived. His work includes the Mona Lisa and The Last Supper are celebrated worldwide. His talents expand beyond his art and his contributions to disciplines such as engineering, astronomy, geometry, botany, geology have revealed is him as a polymath. One discipline where he devoted a considerable amount of time and effort was anatomy. His anatomical notes and drawings can be paralleled to texts books of today. This review explores Leonardo da Vinci’s significant contributions to the study of human anatomy, with a particular focus on reproductive anatomy. Leonardo’s meticulous dissections and detailed anatomical sketches, despite being influenced by the limited scientific understanding and constraints of his time, reveal his deep curiosity and innovative approach. His work, though not widely recognised during his lifetime, laid foundational insights into neuroanatomy, the cardiovascular system, and foetal development. He had a particular interest in reproduction and the genitourinary anatomy and made many anatomical studies in this area. The review also examines the challenges Leonardo faced, such as the lack of preservation techniques and the influence of Galenic teachings, which led to both groundbreaking observations and notable inaccuracies in his anatomical studies or the reproductive system.

**Supplementary Information:**

The online version contains supplementary material available at 10.1007/s43032-024-01772-9.

## Introduction

**Fig. 1:**
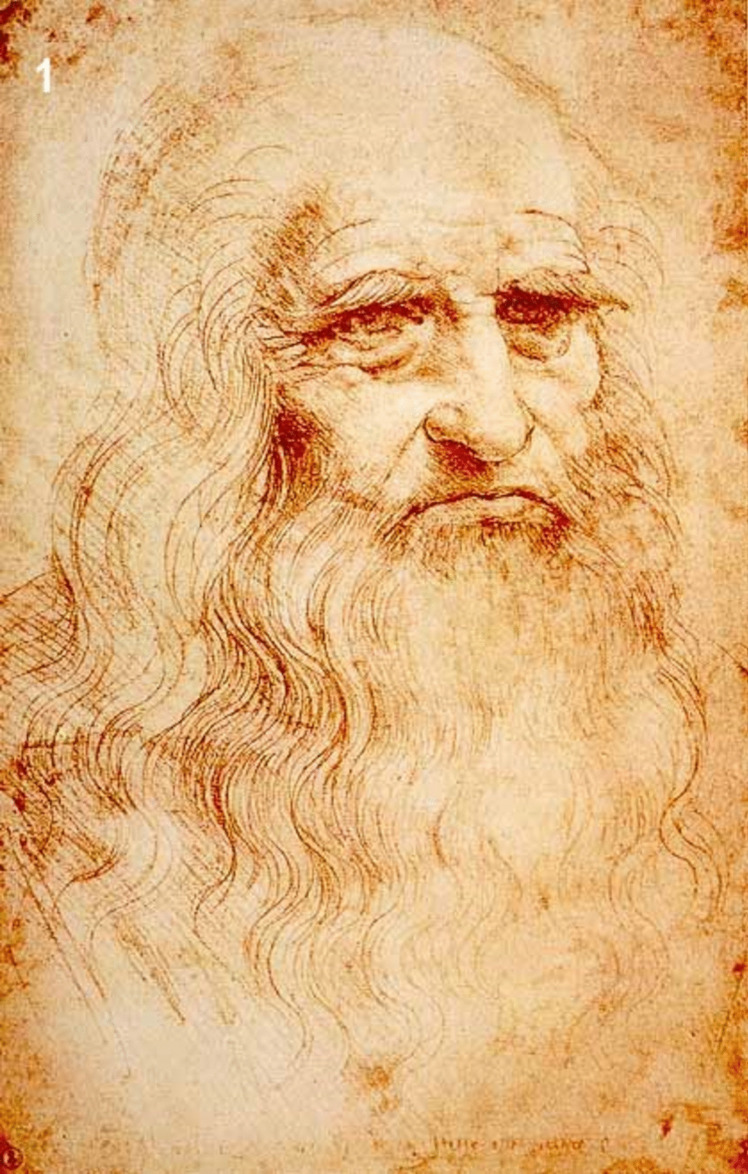
Portrate of Leonardo de Vinci (1452–1519) en.wikipedia.org/wiki/Leonardo_da_Vinci

Leonardo da Vinci (Fig. 1) is regarded as one of the greatest artists who ever lived and was considered a multitalented polymath in his own lifetime. He carried out in-depth studies in areas such as optics, physics, geology and hydrology, astronomy, engineering, and anatomy with many of his insights being centuries ahead of his time [1]. He was born in Anchiano, a small hamlet near Vinci close to the city of Florence on the 15th of April 1452 to a Caterina di Meo Lippi, a 16-year-old peasant girl, and ser Piero da Vinci, a 26-year-old notary. As he was born out of wedlock, it is thought that he was raised with his paternal grandparents. Furthermore, being illegitimate, he was barred from pursuing a career as a notary and thus received a basic education in reading, writing, and arithmetic [2]. This worked out to his advantage – liberating him from the constraints and monotony of an officiate and allowing him the freedom to be creative.

At the age of 14, his father secured him an apprenticeship in Andrea del Verrocchio’s bottega in Florence. Verrocchio was a renowned painter and sculptor, and with him, Leonardo was exposed to theoretical ideas and technical skills in art. During Leonardo’s apprenticeship and throughout his early years in Florence, he developed as a full-fledged artist, completing the requirements to join the confraternity of the Florentine guild of painters. His talents, genius, and affable demeanour attracted the attention of notable people of the time [3]. He also had professional relationships with other prominent figures, including Niccolò Machiavelli. He moved from Florence and lived for some duration of his life in Milan, Rome, and finally France. His patrons included the Medici family, Ludovico Sforza, Cesare Borgia, and lastly, King Francis I of France [2]. These patronages allowed him the freedom to conduct his investigations in many disciplines he was interested in.

Leonardo’s initial exposure to anatomy occurred during his time at Verrocchio’s bottega, where studying the human form was crucial for achieving realistic depictions. Producing such detailed anatomical drawings demanded not only precise sketching skills but also the ability to meticulously dissect and accurately represent the structures being observed. As Leonardo’s enthusiasm for dissection deepened, it drove him to pursue anatomy as a discipline to explore with more intrigue.

## Leonardo’s Anatomical Work

The commencement of systematic human cadaveric dissections in the 14th century facilitated the advancement of medical science, and quickly spread to cities in northern Italy [4]. Leonardo’s position on the practice of human dissections is expressed in his notes:

*You should not be distressed that your discoveries come from another’s death – rather you should rejoice that our Creator has provided an instrument of such excellence* [5].

During his lifetime, Leonardo may have dissected around 30 human corpses [6, 7], including a centenarian, a 2-year-old child, and a 4-month-old fetus [8]. His dissections were mainly carried out in the hospital of Santa Maria Nuova in Florence, and later in Santo Spirito Hospital in Rome [7].

According to his biographer, Vasari,

“*he was one of the first that began to illustrate the problems of medicine with the doctrine of Galen, and to throw true light on anatomy, which up to that time had been veiled in the thick and gross shadow of ignorance. And in this he found marvellous aid in the brain, work, and hand of Leonardo, who made a sketchbook with drawings in red chalk retouched in pen and ink: the bodies that he dissected with his own hand were drawn with the greatest diligence*” [3].

Leonardo understood the value of anatomical drawings as superior instructional learning against those who argue that watching an anatomist at work is more instructive. He acknowledged that observing a live dissection can be valuable, but he insisted that his detailed drawings offer insights that cannot be gained from observing a single dissection.

His preliminary anatomical studies were inspired by the writings of Galen and Avicenna [9]. He held Galen’s writings in high regard and utilised some of his dissection techniques on apes [10]. Leonardo also read the work of the noted surgeon and anatomist, Mondino de’ Liuzzi (1275–1326), who practiced public dissections of human cadavers in Bologna, publishing the first modern anatomical text *Anathomia Corporis Humani* [10, 9].

Leonardo’s anatomical studies span over three decades and can be apportioned into three main phases as outlined by Wells [11].

- First Period (Early 1480s to 1495 in Milan):

During this time, Leonardo’s work shows early influence from Galenic and Hippocratic ideas, such as primitive notions of circulation and procreation. Noteworthy are his advanced and accurate depictions of the skull’s bony structure, which were significantly ahead of his time. Despite some misconceptions (e.g., procreation), his skull drawings stand out for their originality and insight.

- Second Period (1506–1509, between Milan and Florence):

This phase includes his dissection of the centenarian in Florence around 1507–1508, which Leonardo meticulously documented. This dissection led to the first recorded description of arteriosclerosis and liver cirrhosis. Leonardo’s work during this period marks a transition from strict Galenic teachings to more independent, original thinking. His studies, particularly of the mesenteric arteries and veins, reflect this intellectual evolution.

- Third Period (1510–1516, in Milan, Pavia, and Rome):

During this time, Leonardo worked with anatomist Marcantonio della Torre, who was the chair of Medicine of the University of Pavia and professor of anatomy [12].

The account of which is noted by Vasari:

*Leonardo then applied himself, but with even greater care, to the study of human anatomy, working together with Messer Marc’Antonio della Torre, an excellent philosopher, who was then lecturing in Pavia and writing on the subject; he was one of the first (as I have heard it said) who, with Galen’s teachings, began to bring honour to medical studies and to shed real light upon anatomy, which had until that time been shrouded in the deepest shadows of ignorance. In this work, he was marvellously served by the genius, labour, and hand of Leonardo, who created a book with red crayon drawings outlined in pen in which he sketched cadavers he had dissected with his own hand, depicting them with the greatest care* [3].

Through this collaboration, he was able to focus extensively on the heart, respiratory system, and other organ systems. Leonardo’s studies included detailed observations of the bronchial arteries, diaphragm, and larynx [6]. His anatomical work culminated in extraordinary depictions of the heart and a notable drawing of the female body (Fig. 12), blending human and animal anatomy, reflecting the continuing influence of Galenic practice [9]. It has been proposed that this collaboration with Marcantonio might have resulted in the completion of his anatomy book. In 1510, he writes in his notes:

*This winter I will finish all this anatomy* [5].

## Leonardo da Vinci’s Reproductive Anatomy

### Male Reproductive Anatomy

**Fig. 2:**
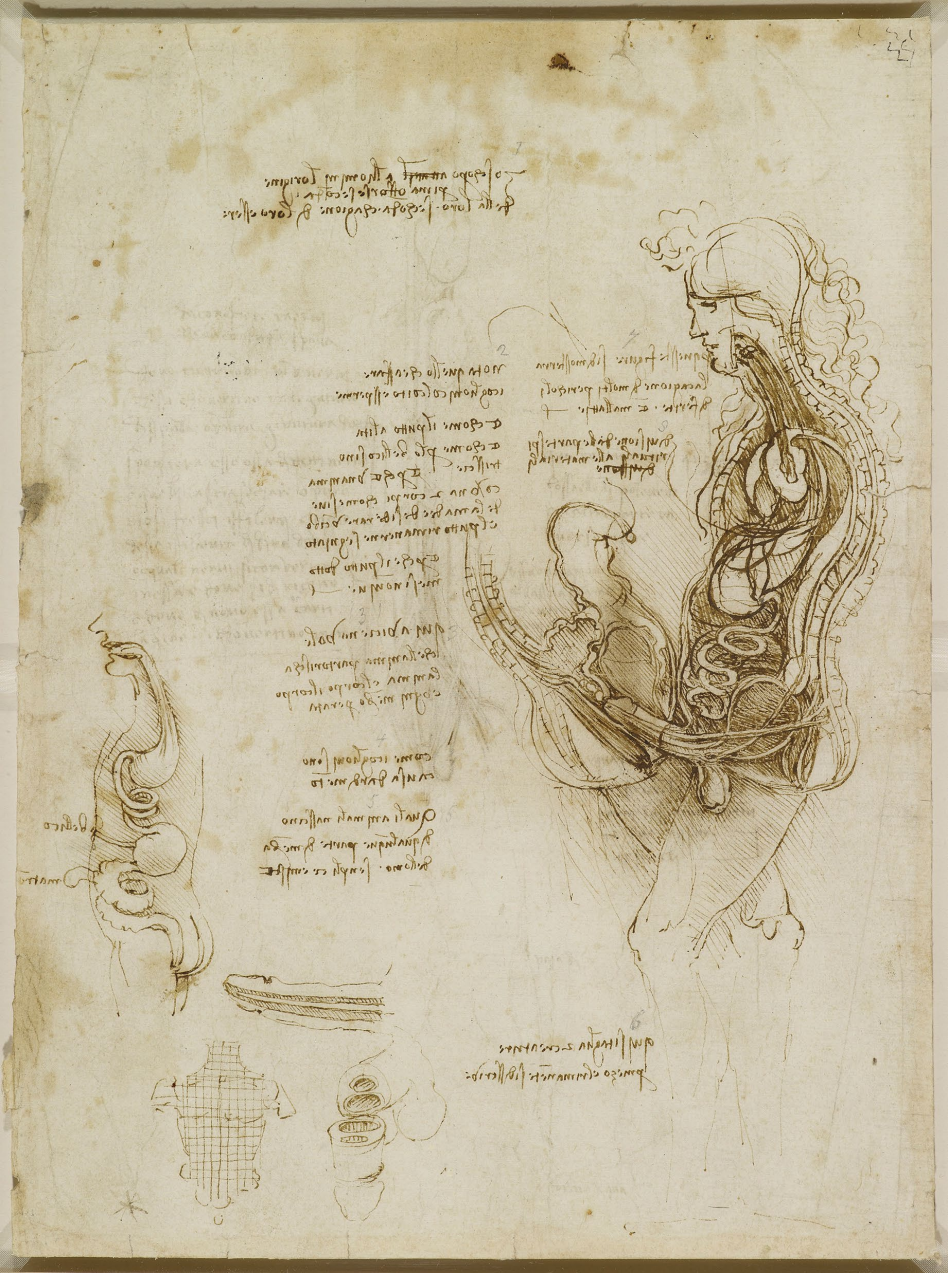
The Hemisection of a man and woman in the act of coition c.1490-92 Pen and ink, 27.6 x 20.4 cm (sheet of paper), RCIN 919097, (with permission from The Royal Collection Trust)

One of Leonardo da Vinci’s first depictions of the man’s reproductive organs is seen in The Hemisection of a Man and Woman in the Act of Coition (c.1490–92), Fig. 2. In this sketching, done in pen and ink, the act of coitus is described in a sagittal view with the penis inserted into the vagina closely touching the uterine cervix. In his notes he writes:

*I display to men the origins of their second – first or perhaps second cause of existence* [5].

There appears to be droplets of semen ejaculated from the penis into the internal os of the cervix. He draws three channels into the penis: from the heart, at the lower end of the spinal column, and from the testis. The channels from heart and testes merge with the urethra and pass through one duct in the penis. The spinal column channel originates from a network of three - merging into a single, separate penile duct. He further emphasises the double penile channels in the drawings bottom centre of the page. A sagittal section, clearly showing two ducts and a cross-section with the same depiction. The upper channel may be the dorsal vein of the penis, which Leonardo misconstrued [11].

**Fig. 3:**
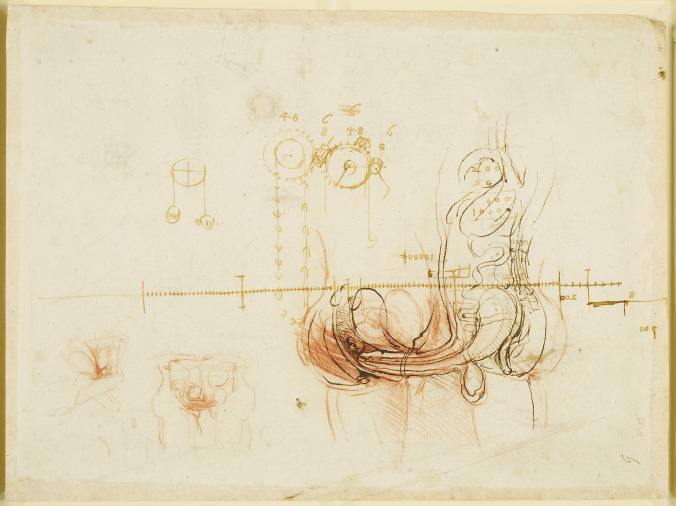
Anatomical studies of the act of coitus, c. 1490-93, Pen and ink and red chalk, 21.3 x 28.5 cm (sheet of paper), RCIN 919096 (with permission from The Royal Collection Trust)

In his sketching of The Coition of a Man & a Woman (Fig. 3), he shows the penis inserted in the vagina. In this drawing, the penis includes a single urethra, a contrast to his double penile ducts from Fig. 2. A further two drawings of the act of coitus are seen on the bottom left of the page. In the reverse page, there are sketches of male and female organs in coition. One appears to depict ejaculation. Included in these pages are sketches of weights and wheels, calculations, drawings of machinery for the excavation of canals, and a topographical sketch of a canal stemming out of a winding river.

**Fig. 4:**
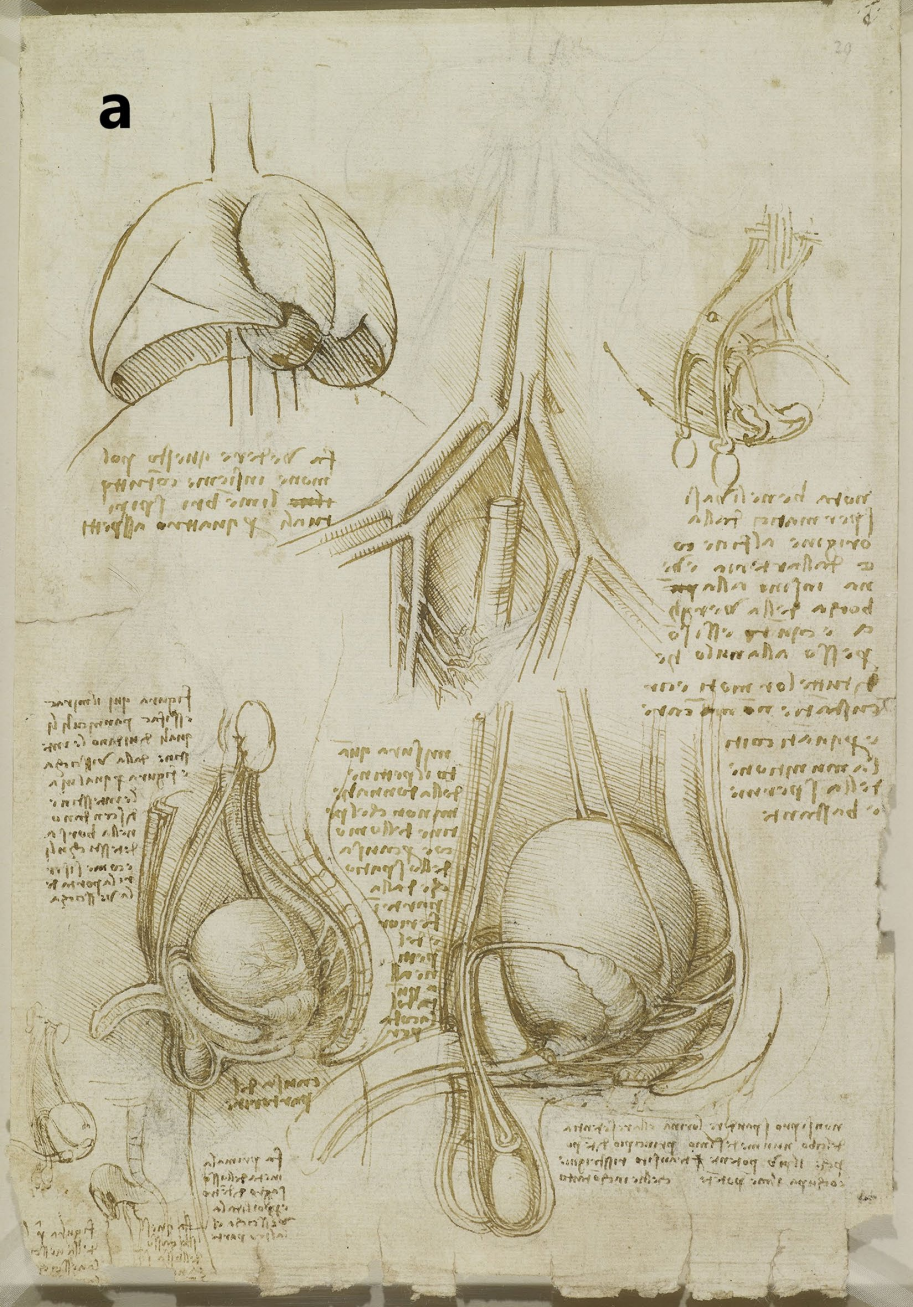

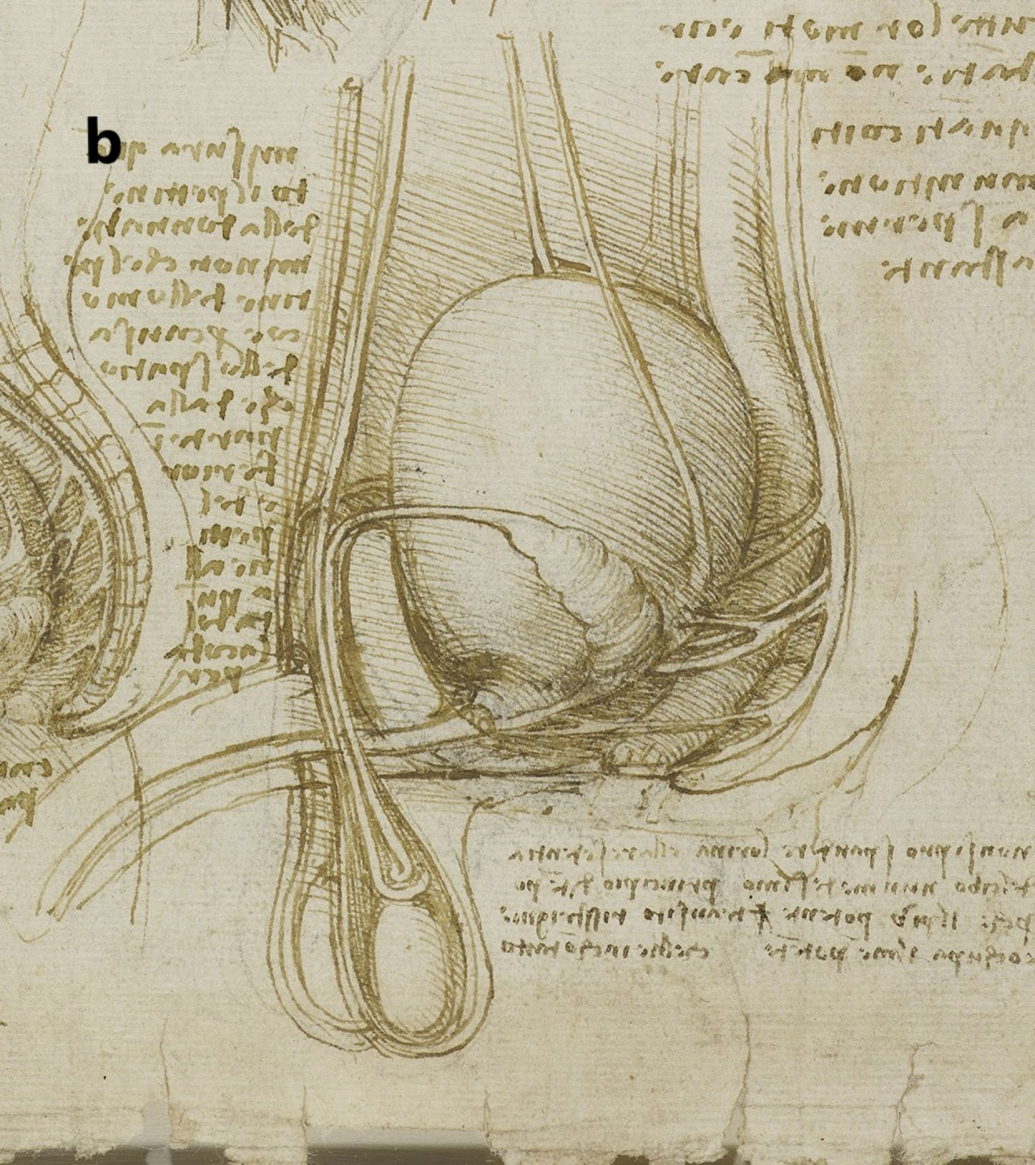

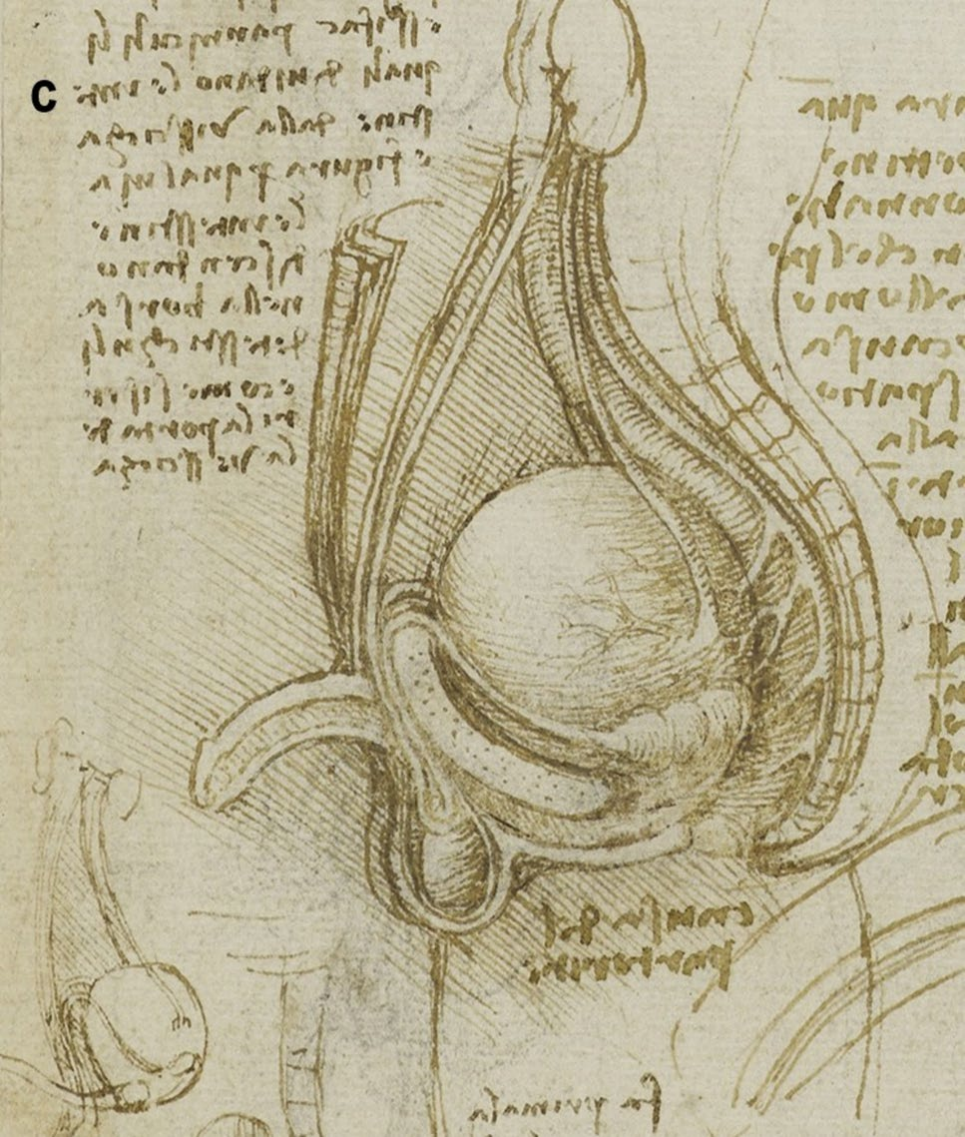
**(a)** The male genitourinary system c. 1508, Pen ink over black chalk, 27.2 x 19.2 cm (sheet of paper), RCIN 919098 (with permission from The Royal Collection Trust) **(b)** Enlarged image - The male genitourinary system c. 1508, RCIN 919098 (with permission from The Royal Collection Trust) **(c)** Enlarged image - The male genitourinary system c. 1508, RCIN 919098 (with permission from The Royal Collection Trust)

A more detailed study of the male reproductive organs is found in the pages of The Male Genitourinary System (Fig. 4a). On the bottom of the page are two large drawings where Leonardo describes the organs of the genitourinary system. In both drawings, the ureters descend from the kidneys to an (oversized) bladder. The larger drawing (Fig. 4b) on the right shows a thin spermatic chord descending from the upper abdomen ending in a coil on top of the testis. There lacks any detail of the network of blood vesicles that form the pampiniform plexus. This vesicle likely represents the testicular artery. The vas deferens ascends from the testis and circumvents the pubis, joining a large seminal vesicle as the ejaculatory duct and continuing around the end of the bladder, forming a single urethra. Two small circles are drawn at the base of the bladder—one clearly representing the lumen of the ejaculatory duct coming from the seminal vesicle—the other circle likely representing the other duct. This was a keen observation from Leonardo showing where these ducts enter the urethra inferior to the fundus of the bladder. His insights are demonstrated in his notes:

*…see which is the first in the urinary canal either the mouths of the spermatic vessel or the mouth of the urinary vessel. But I believe that the urine is first so that it can then clean and wash out the sperm which makes the urinary canal sticky* [5].

It is noted, however, that the prostate gland is not included in any of his drawings of the male reproductive system. The testis is positioned in the scrotal sack, it is ovoid in shape surrounded by a fluid-filled cavity. The epididymis is not drawn or misrepresented as some other vesicles. The smaller drawing to the right (Fig. 4c) of the page shows the testicular arteries branching off the aorta and passing (just beneath the kidneys) as with the larger drawing to the right – it ends in a coil on top of the testis. The vas deferens can be seen arising from the testis – looping over an oversized pubis and joining the seminal vesicle. The ejaculatory duct is joining a large single urethra. A network of channels is drawn connecting the spinal column with the seminal vesicle, sperm duct, and bladder.

**Fig. 5:**
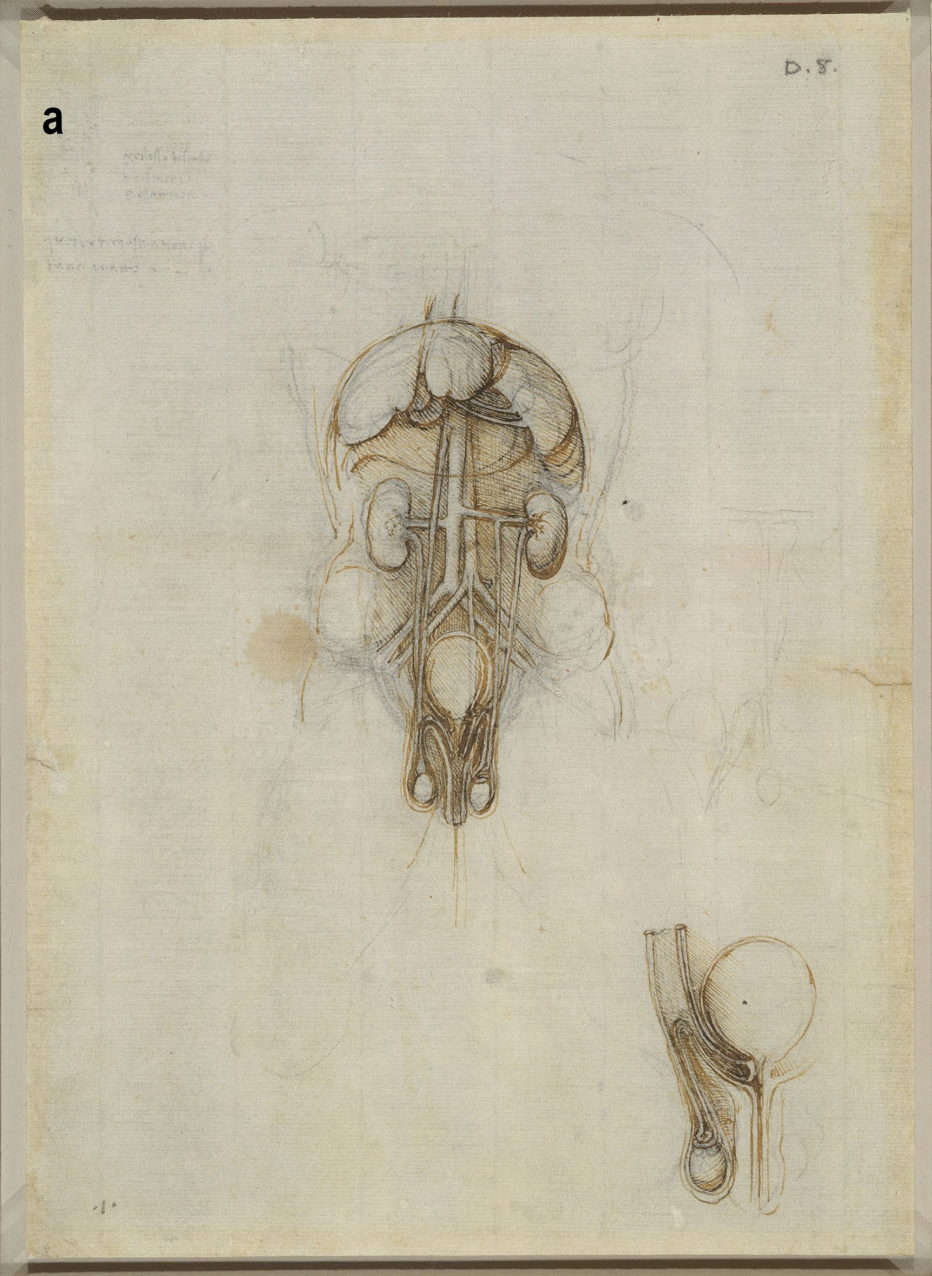

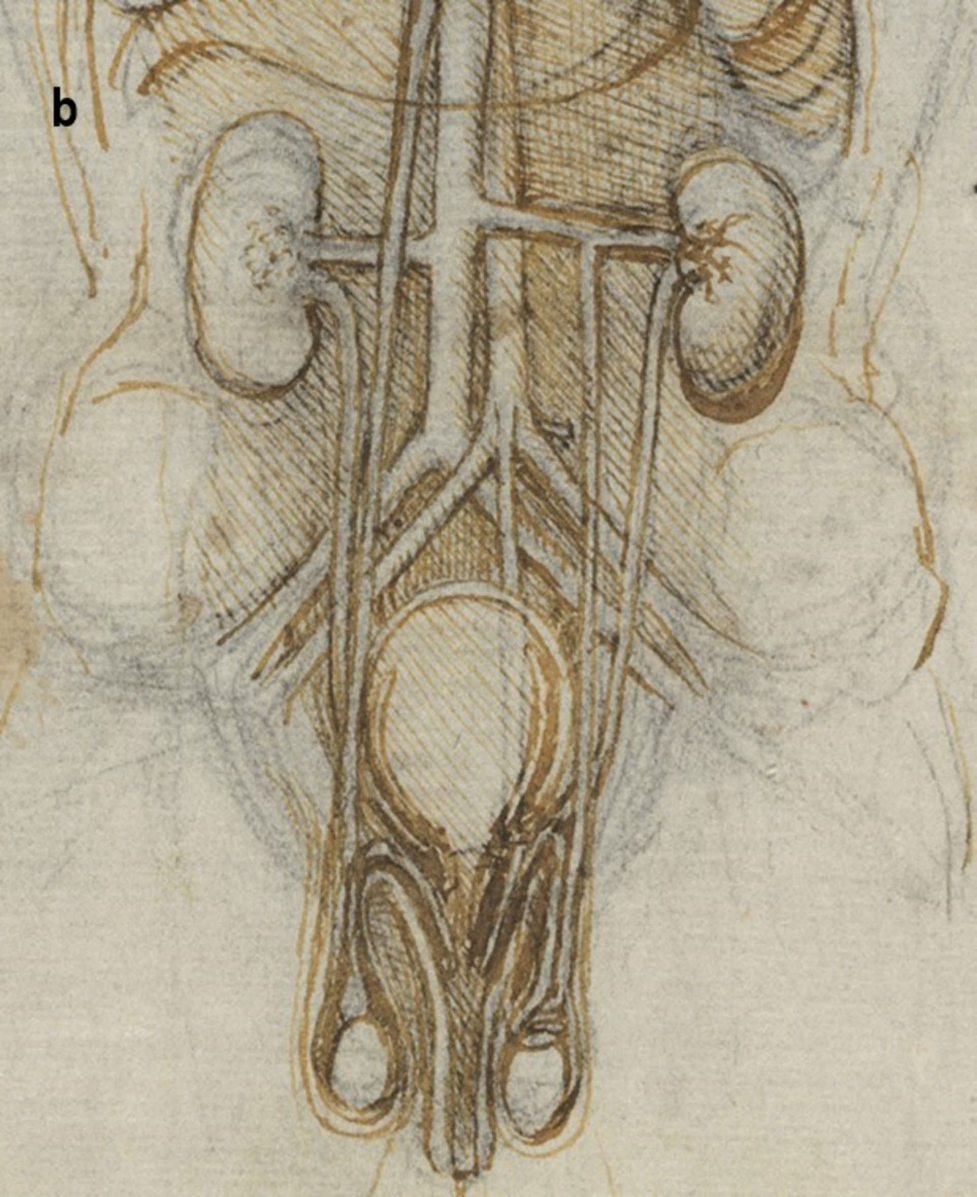

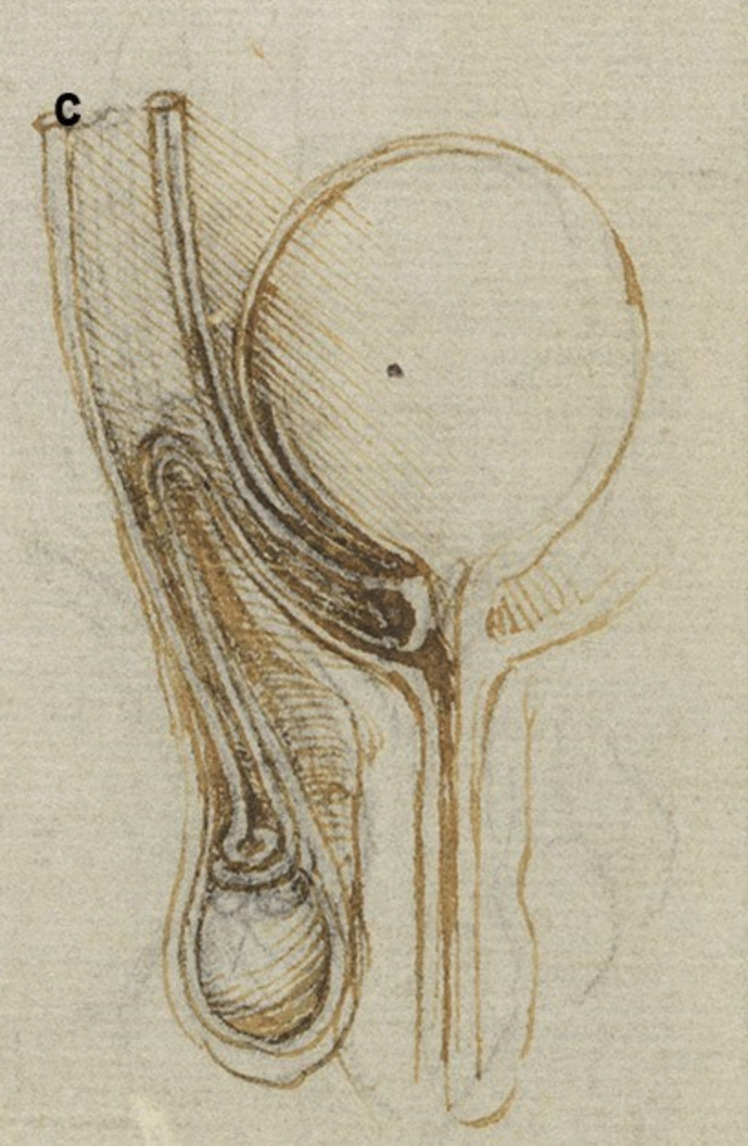
**(a)** Anterior view of male torso showing genitourinary system, liver, spleen & stomach; sketch illustrating principles of fertilisation; bladder, penis, & R ureter & testis, Pen and ink over traces of black chalk, 26.3 x 18.9 cm (sheet of paper), RCIN 919099 (with permission from The Royal Collection Trust) **(b)** Enlarged image - genitourinary system, RCIN 919099 (with permission from The Royal Collection Trust) **(c)** Enlarged image - genitourinary system, RCIN 919099 (with permission from The Royal Collection Trust)

Leonardo’s interests in the male genitourinary system are further demonstrated in Fig. 5a. On the drawing centre of the page (enlarged Fig. 5b) - the testicular arteries can be seen branching from the aorta. However, the left testicular artery branches from the renal artery, whereas the right testicular artery originates further up the aorta just below the liver. Both testicular arteries and vas deferens end in coil on the testis. The vas deferens is shown to loop and form a single urethra. The drawing bottom right (enlarged Fig. 5c) shows the right testis in situ with minimal scrotal space. The testicular artery and vas deferens form a large coil on top of the testis. It is possible this structure may be the epididymis.

**Fig. 6:**
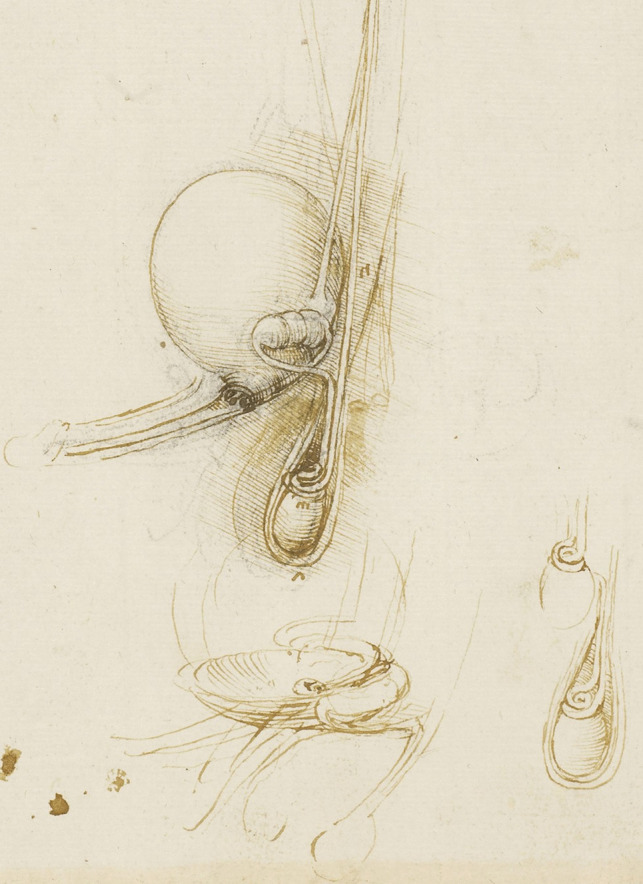
The male genitourinary system c.1508-10, Pen and ink over black chalk, 27.0 x 18.9 cm (sheet of paper), RCIN 919100 (with permission from The Royal Collection Trust)

In the drawings depicted in Fig. 6, there is an illustration of an expanded, full bladder along with the ureter (the sketch immediately beneath this drawing seems to depict an empty bladder, deflated into a concave shape). The scrotum, testis, and vas deferens are labelled A, M, and N respectively. The vas deferens ascends and joins the seminal vesicle merging what appears through the bladder sphincter joining the urethra. A more detailed sketch of the testis and spermatic chord is illustrated bottom right. Here, it appears that the coils drawn represent the epididymis. These details are an advance from the testis drawn in Fig. 5a.

Through further investigation, Leonardo’s understanding of the anatomy and physiology of the penis grew, disputing some of the Hippocratic-Galenic notions. He posited, correctly, that penile erection (tumescence) was not brought about by pneuma (accumulation of air) and vital spirits flowing into the penis as proposed by Hippocrates and later by Galen [13]. He argued that such a notion was physically implausible, as the amount of air required to create the density and rigidity of an erect penis would not be sufficient. Furthermore, in 1477, he was able to observe the dissection of hanged men and noted:

“*I have seen. .. dead men who have the member erected, for many die thus, especially those hanged…..I have seen the anatomy, all of them having great density and hardness, and being quite filled by a large quantity of blood* [13].

Through his keen observation, he was one of the first to describe the cause of tumescence during which the penis becomes engorged with blood.

Leonardo was also aware of the nature of penile erections and how they are unpredictable and autonomous - likening it to a separate entity with its *own will and intellect*. He describes erections occurring while sleeping and how can act contrary to man’s desires—being active when a man wishes it inactive, and vice versa. He also questions why it is a source of shame and embarrassment, hidden and concealed, rather than celebrated and openly acknowledged [14]. This demonstrates his blend of anatomical and physiological observations with cultural reflections.

## Comparison of Anatomical Details

**Fig. 7:**
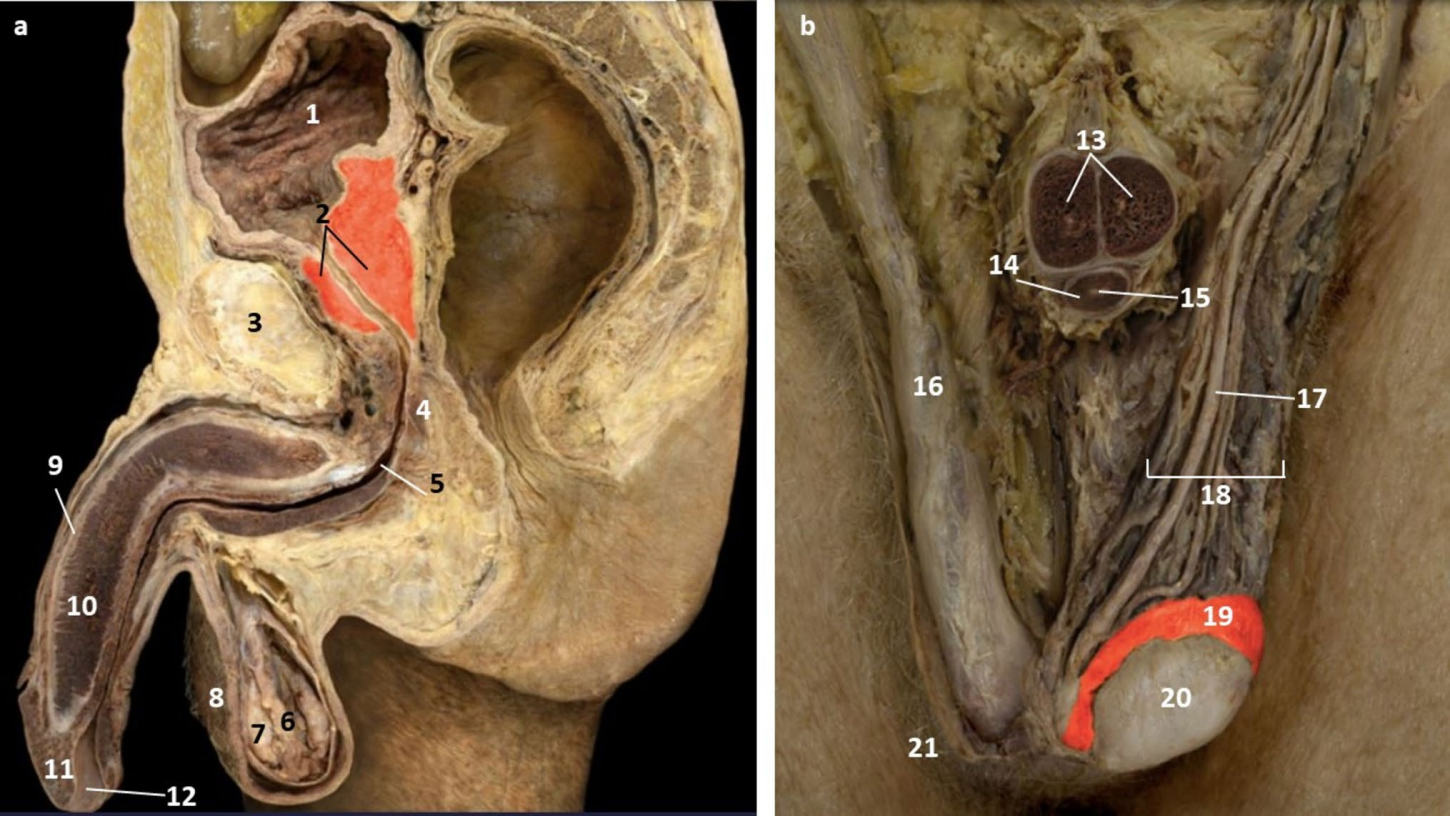
The male genitourinary system (curtesy of Shane McTauge, University of Iowa). **a**. Bladder, 2: Prostate gland, 3: Pubis (Symphysis pubica), 4: seminal vesicle, 5: Urethra, 6: Testis, 7: Epididymis, 8: Scrotum, 9: Penis, 10: Corpus cavernosum, 11: Glans of penis, 12: Urethral meatus. **b**. 13: Corpus cavernosum, 14: Corpus spongiosum, 15: Spongy (penile) urethra, 16: Spermatic cord, 17: Ductus (vas) deferens 18: Pampiniform venous plexus; 19: Epididymis, 20: Testis, 21: Scrotum

In the cadaveric images (Figs. 7a and 7b), the male reproductive organs are distinctly visible, including the penis, scrotum, testis, vas deferens, prostate gland, seminal vesicles, and urethra. The anatomical details are precise, showing the actual relationships and positions of these organs within the body. Leonardo’s sketches reflect a mixture of accurate observation and speculative elements. For instance, in The Hemisection of a Man and Woman in the Act of Coition (Fig. 2), Leonardo depicted the penis with three channels—two ducts for semen and one for urine—stemming from different origins, including the heart and spine, which we now know to be incorrect. This network of channels can also be seen in the drawing of the male reproductive organs (Fig. 4b). These channels emanating from the spine may be the inferior hypogastric plexus (or pelvic plexus), which is formed from the fusion of the pelvic splanchnic nerves, sacral splanchnic nerves, superior hypogastric plexus, and visceral afferent fibres. The hypogastric plexus contains both parasympathetic and sympathetic nervous fibres and supplies the pelvic and perineal organs [15]. They are not involved in providing regenerative energy from Leonardo’s adherence to the writings of Galen and Avicenna [9]. However, his accomplishments in neuroanatomy continue to be recognised [16].

In the cadaveric image (Fig. 7b, 17), the vas deferens is clearly seen emerging from the testis. The spermatic cord is also depicted (Fig. 7b, 16), and with further dissection, the network of vesicles that form the pampiniform plexus becomes obvious (Fig. 7b, 18). These details are absent from Leonardo’s work. However, Leonardo’s depictions of the vas deferens and spermatic cord, particularly in his later drawings, show an evolving understanding, where he began to depict the vas deferens more accurately as looping and joining the seminal vesicle. Moreover, the exaggerated coils he included, which might represent the epididymis or another structure, reflect his exploration and attempts to understand the complexity of these connections. It is, however, obvious that the prostate gland, clearly observed in the cadaveric image (Figs. 7a, 2), is absent from Leonardo’s drawings. This omission may be due to him not drawing from the specimens directly – but from his contemplation of a mixture of animal and human observations. In the cadaveric images (Figs. 7a and 7b), the testis is clearly visible within the scrotum, and the epididymis can also be discerned, though it does not display the highly coiled structure found in Leonardo’s illustrations. A more detailed view of the epididymis is shown in Fig. 7b, 19.

In Leonardo’s later drawings (Figs. 5b, c, and 6), the epididymis is likely represented by the coiled structure on top of the testis. However, his depiction lacks the detailed accuracy of modern anatomical understanding, where the epididymis is a more clearly defined structure adjacent to the testis (Fig. 7b, 19).

## Female Reproductive Anatomy

Leonardo’s depiction of the female reproductive anatomy can also be observed in The Hemisection of a Man and Woman in the Act of Coition (Fig. 2) – where he draws the uterus, cervix, and vagina. In this figure, he draws the spine diverging, with one branch of the spinal cord passing directly into the uterus. The uterus is crenated with a duct passing towards the breast and connecting at the nipple. Leonardo’s notions of reproduction may have adhered to the traditional notions of reproduction and procreation proposed by Hippocrates (450—380 BCE), Galen (129–199 A.D.), and Avicenna (Ibn Sina, 980–1037 A.D.) [9].

Hippocrates, often referred to as the "Father of Medicine," laid the foundation for the understanding of reproduction with his theory of the four humors—blood, phlegm, yellow bile, and black bile [17]. He believed that the balance of these humors was essential for health, including reproductive health [18]. According to Hippocrates, both men and women contributed to reproduction through the release of seed, with the health and balance of the humors affecting fertility and the development of the embryo [19]. Galen expanded on Hippocratic ideas, further developing the concept that both sexes contributed equally to reproduction. He theorised that both men and women produced "seeds," which combined during conception, with the male seed being more active in shaping the offspring. Galen also emphasised the role of the uterus, describing it as a vital organ in nurturing the embryo (which may explain the drawing of the duct from the breast to the uterus, Fig. 2). Avicenna, a Persian polymath, synthesised and expanded upon the ideas of Hippocrates and Galen in his seminal work, The Canon of Medicine [17]. Avicenna’s contributions to reproductive theory included detailed descriptions of the anatomy and physiology of the reproductive organs, drawing heavily from Galenic theory [20]. It is evident that Leonardo was aware of the writings of Avicenna, as in his notes he writes:

*Avicenna claims that the soul begets the soul, and the body the body* [5].

Therefore, this image of the act of coitus illustrates traditional beliefs: a second channel in the penis was thought to carry ‘animal spirit’ (loosely interpreted as the soul) from the spinal cord, while a bifurcated spinal cord in the woman was believed to transmit her ‘animal spirit’ to the uterus. It was commonly thought that conception involved both material and spiritual elements. Additionally, a vessel was believed to connect the testes, the source of passion (from his notes: *How the testis are a cause of ardor*, [5]), to the heart, where emotions are experienced, and a vessel from the uterus to the breasts was thought to convert retained menstrual blood into milk after conception.

**Fig. 8:**
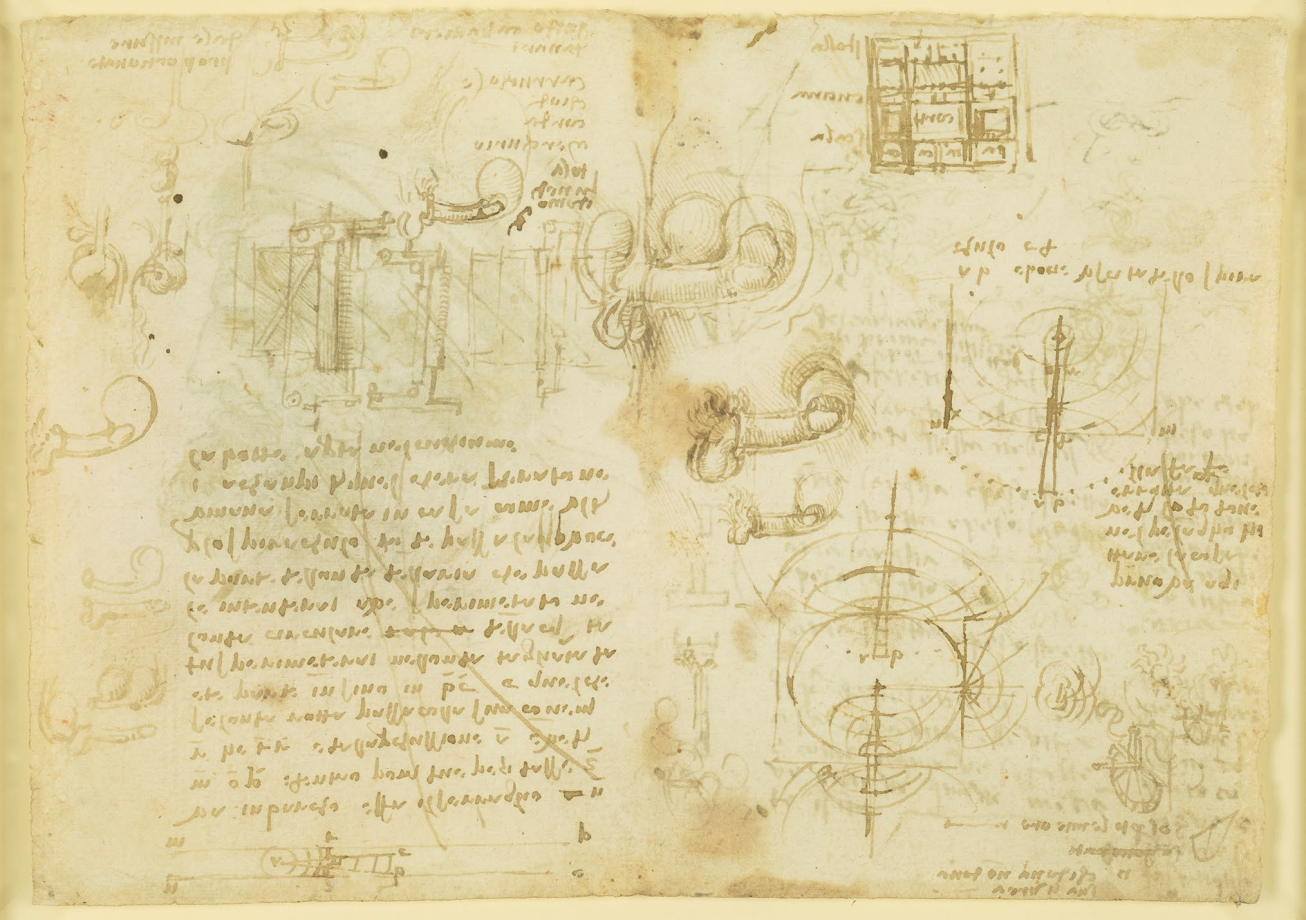
Studies of coition, wave propagation, architecture, engineering, etc. c. 1510, Pen and ink | 14.6 x 20.9 cm (sheet of paper), RCIN 919106 (with permission from The Royal Collection Trust)

Leonardo further describes the act of coition in several other drawings (Fig. 8). In this series of images, Leonardo draws the penis inserted into the vagina and penetrating the cervix and uterine cavity. However, despite his scientific interest, his contemplations and distaste for the sexual act are displayed in the notes contained within the Codex Atlanticus, where he writes:

*’The act of coupling and the members engaged in it are so ugly that if it were not for the faces and the adornment of the actors and the impulses sustained, the human race would die out*’ [1].

**Fig. 9:**
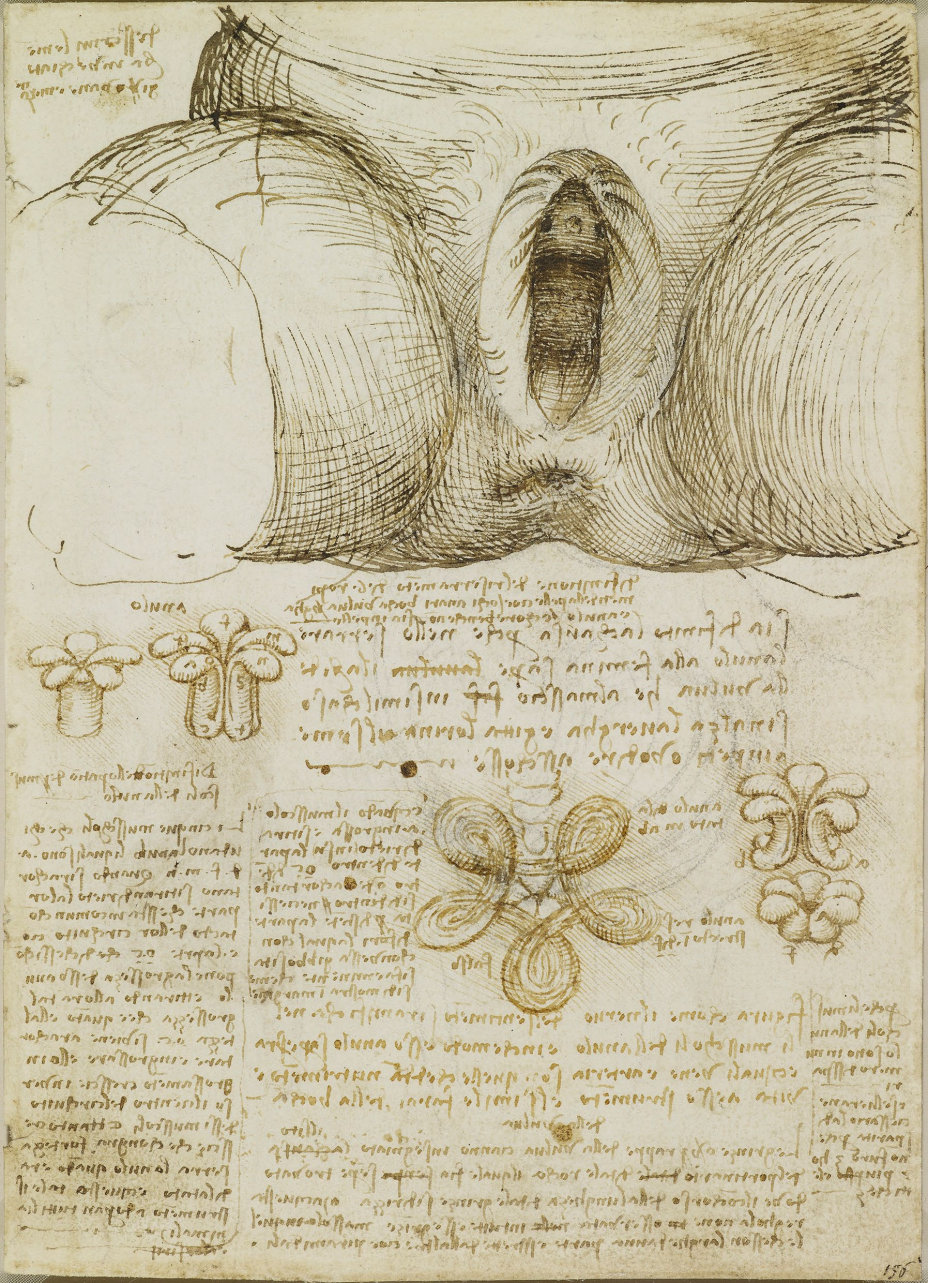
The vulva and anus c.1508 Pen and ink over black chalk, 19.1 x 13.8 cm (sheet of paper), RCIN 919095 (with permission from The Royal Collection Trust)

Leonardo describes the external genitalia and vagina of a multiparous woman (Fig. 9). He depicts the vestibule and urethral orifice. The vaginal introitus is obvious. However, the labia minor and clitoris are absent. His accompanying sketches describe his notions on the mechanism of the anal sphincter.

**Fig. 10:**
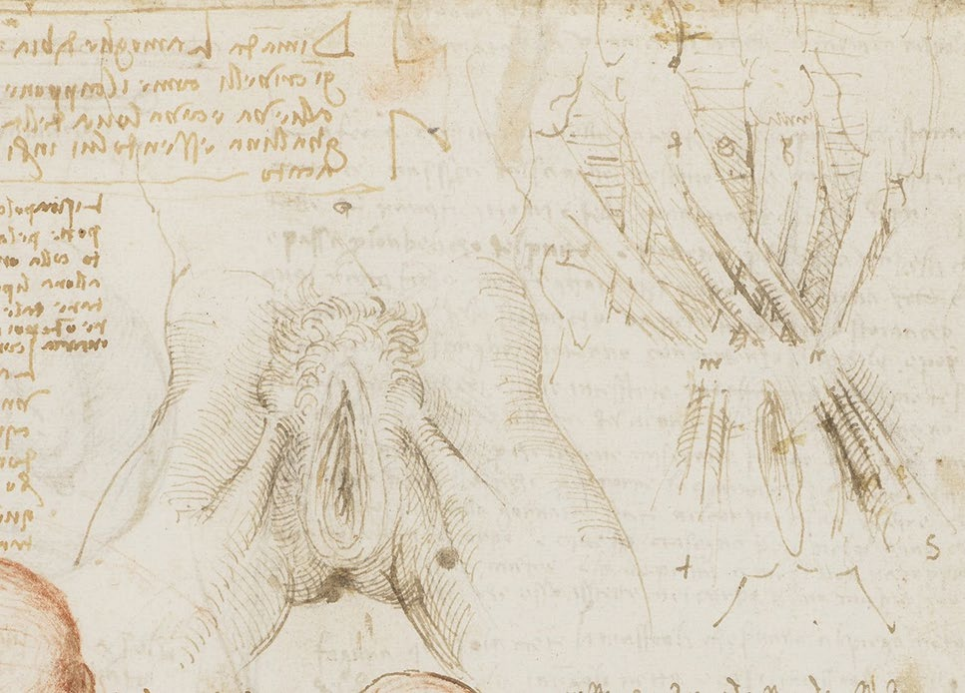
The fetus, and the muscles attached to the pelvis Red and black chalks, pen and ink, wash. 30.4 x 21.3 cm (sheet of paper), RCIN 919101 (with permission from The Royal Collection Trust)

Figure 10 shows a more detailed drawing of the external genitalia of a woman. In this drawing the vulva with labia majora and minora are depicted – with the clitoral prepuce present. His accompanying notes include some further thoughts on sexual desire and anatomical function. He writes:

*The woman commonly has a desire quite the opposite of that of man. This is, that the woman likes the size of the genital member of the man to be as large as possible, and the man desires the opposite in the genital member of the woman, so that neither one nor the other ever attains his interest because Nature, who cannot be blamed, has so provided because of parturition* [21].

**Fig. 11:**
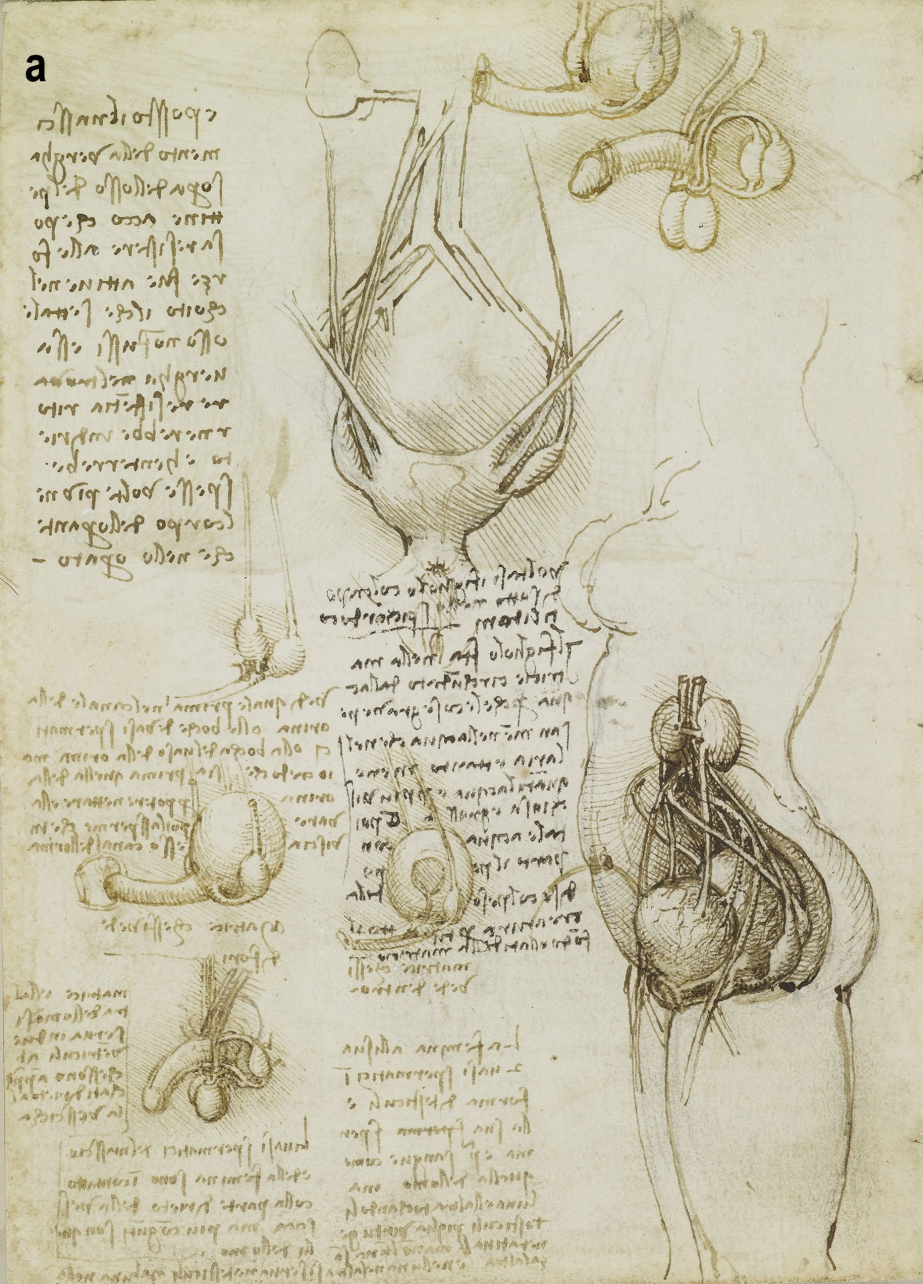

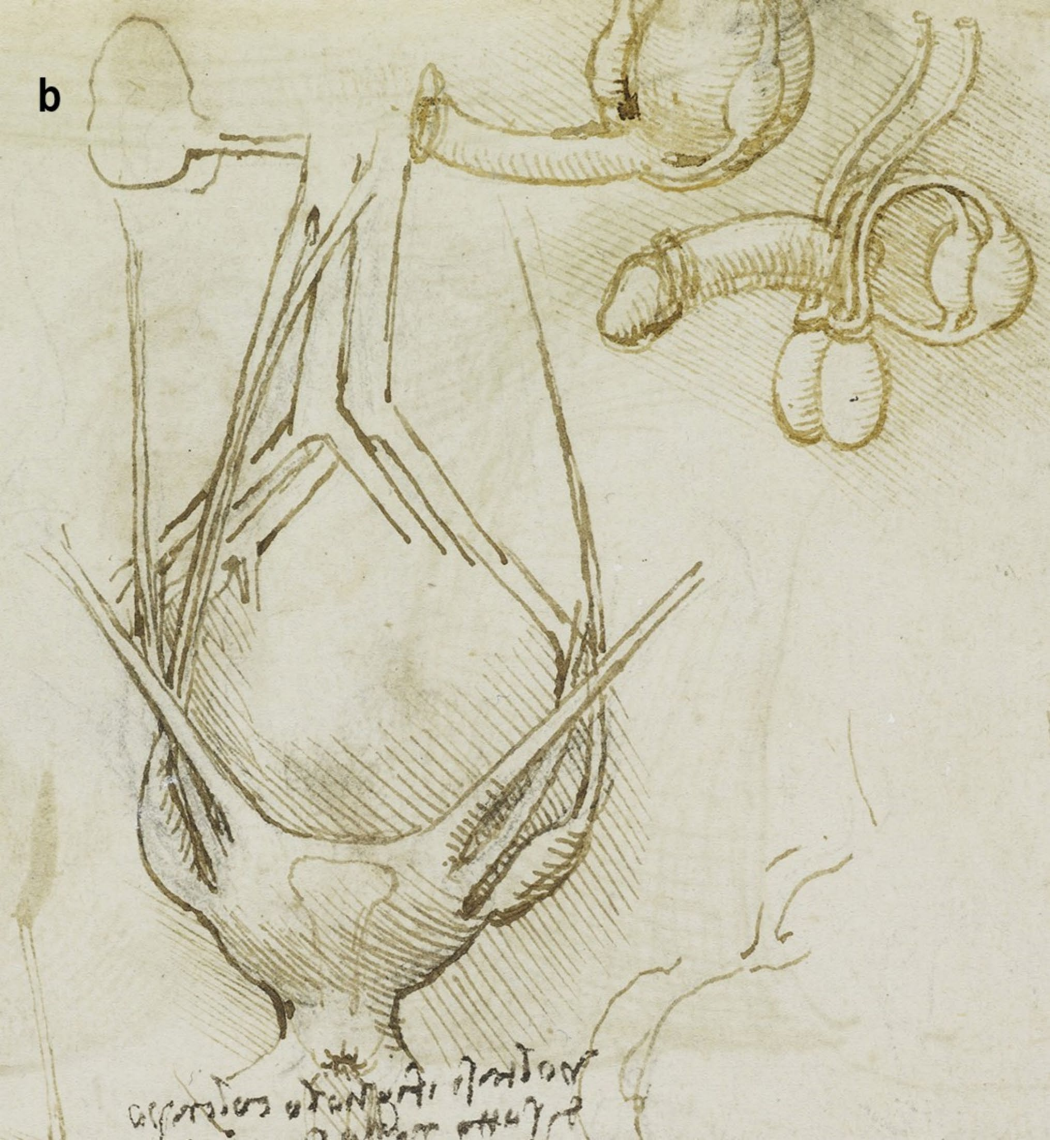

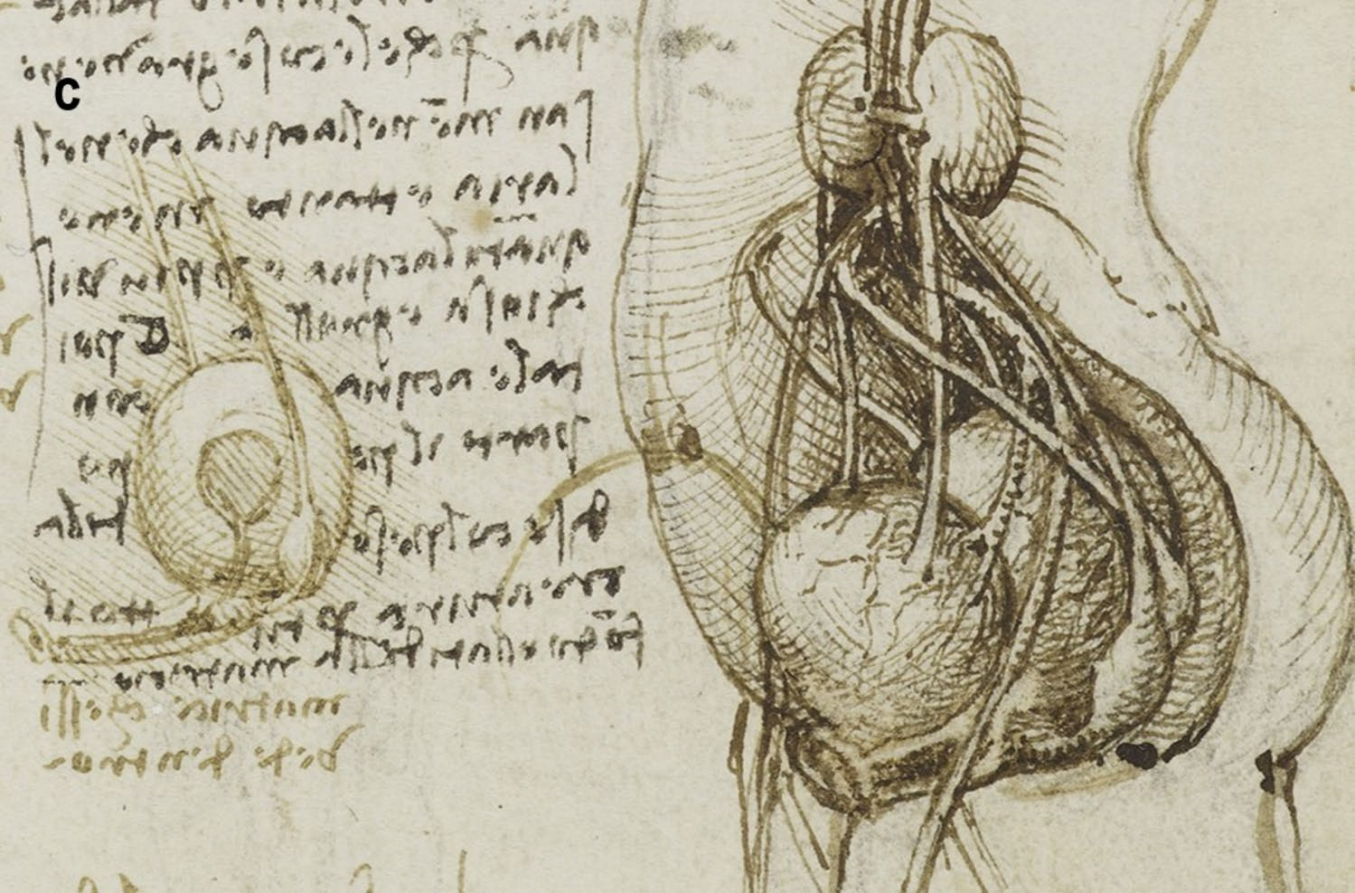
**(a)** The male and female reproductive systems c.1508,Pen and ink over black chalk, 19.1 x 13.8 cm (sheet of paper), RCIN 919095 (with permission from The Royal Collection Trust) **(b)** The male and female reproductive systems enlarged (with permission from The Royal Collection Trust) **(c)** The male and female reproductive systems enlarged (with permission from The Royal Collection Trust)

A more detailed analysis of female reproductive anatomy, along with a comparison to male reproductive anatomy, is depicted in Fig. 11a - The Male and Female Reproductive Systems (c.1508). In this work, the similarities between male and female reproductive organs are examined. These homology studies may have been incentivised following his reading of Avicenna, who depicted the female and male reproductive organs as homologous anatomical structures, with differences only in the location, size, and complexity [20].

In Fig. 11b, the male and female reproductive organs are illustrated side by side. The image at the top shows the vagina and uterus, with the ‘testicles’ (ovaries) on either side. The uterus is depicted as rounded, connected to an elongated, curved vagina. Adhering to the Galenic understanding of reproduction, ducts are drawn to represent the pathways for procreative elements. In his notes, he writes:

*The female has her two spermatic vessels in the form of testicles, and her sperm is initially blood, like that of the male. But in both, upon reaching the testicles, it acquires generative power. In neither case is it stored in the testicles; in the female, it is kept in the uterus, and in the male, in the two ventricles attached to the back of the bladder* [5].

The definitions and more accurate description of the male and female reproductive organs were not established during his time and would have to wait for over a century for the works of Dutch anatomists Nicolaus Steno (1638–1683), Jan Swammerdam (1637–1680), and Reinier de Graaf (1641–1673) [19, 22].

Figure 11b shows a larger drawing of the uterus. Here the cervical os and fundus can be seen. There is a lighter sketch demonstrating the internal uterine cavity (an advance on the earlier notion of a multichambered uterus). The ovaries (or spermatic vessels as he termed them) are drawn close to the uterus on either side. There are minimal Fallopian tubes. However, extending from each ovary are the vessels that join the aorta (which may be the source of the flow of blood to the ovaries as described in his quote above). There are two large projections emanating from the uterine fundus, which likely represent the round ligament of the uterus. On the left of the figure can be seen the ureter descending from the kidney and joining the uterus.

Another figure of the female reproductive (and urinary) system is described in Fig. 11c. To the right is a small sketch of the uterus, showing the uterine cavity, cervix, and vagina. As with Fig. 11b, a large vessel extends from the ovary upwards (joining the aorta). To the left is a more detailed sketch. The ureters extend from the kidneys, attaching to an extended bladder. Positioned directly behind the bladder is the correctly placed uterus. A network of vessels is visible. The ovary is attached near the end of the uterus via a small duct, referred to as the vas sperminarium, which is believed to transport sperm to the ovaries [21]. This is likely the ligament of the ovary. Emerging from the ovaries can be seen the vessels arising to join the aorta. Another large blood vessel, which may represent the iliac artery, can be seen joining the bladder and bifurcating to the external iliac artery.

It has been suggested that this drawing represents a uterus in early pregnancy, and the notes accompanying this page of drawings include:

*The child lies in the uterus surrounded with water because the heavy things weigh less in water than in the air, and the less so the more viscous and greasy the water is. And then such water imparts its own weight with the weight of the creature over the whole bottom and the sides of the uterus* [6].

**Fig. 12:**
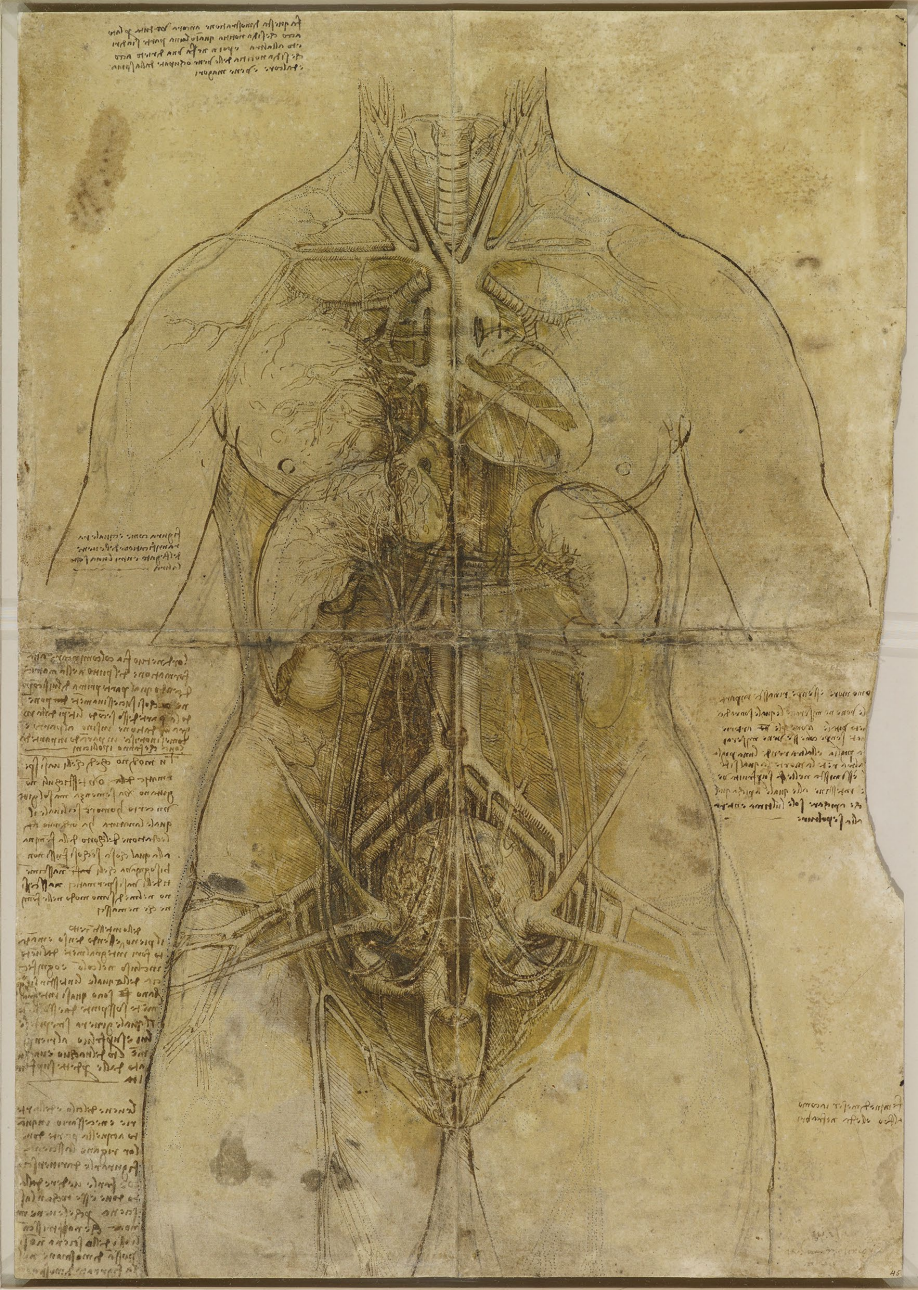
The cardiovascular system and principal organs of a woman c.1509-10, Black and red chalk, pen and ink, yellow wash, on toned paper, pricked through, 47.6 x 33.2 cm (sheet of paper), RCIN 912281 (with permission from The Royal Collection Trust)

In his detailed drawing The Cardiovascular System and Principal Organs of a Woman (c.1509-10) (Fig. 12), Leonardo outlines the relationship of the cardiovascular system and other organs, including the genitourinary system.

The umbilical vein extends diagonally from the umbilicus to the liver, while the umbilical arteries and their presumed venous counterparts loop downward. Although these vessels initially appear continuous with the ureters and ovarian vessels, they connect to the common iliac vessels. The ureters run from the kidneys to the bladder, with no visible urethra and an uncertain connection to the vagina. The right ovarian vessels are accurately depicted, but the left ovarian artery is incorrectly shown originating from the left renal artery instead of the aorta.

The uterus is illustrated as a large, spherical, multichambered structure, with ligaments extending towards the pelvis. The cervix can be seen protruding into the vaginal cavity, forming the ectocervix. Vessels ascend from the uterus, possibly representing those believed to carry retained menses to the breasts. This drawing is a fascinating blend of traditional ideas, adhering to dogma of the period [23] and precise observations.

## Comparison of Anatomical Details

**Fig. 13:**
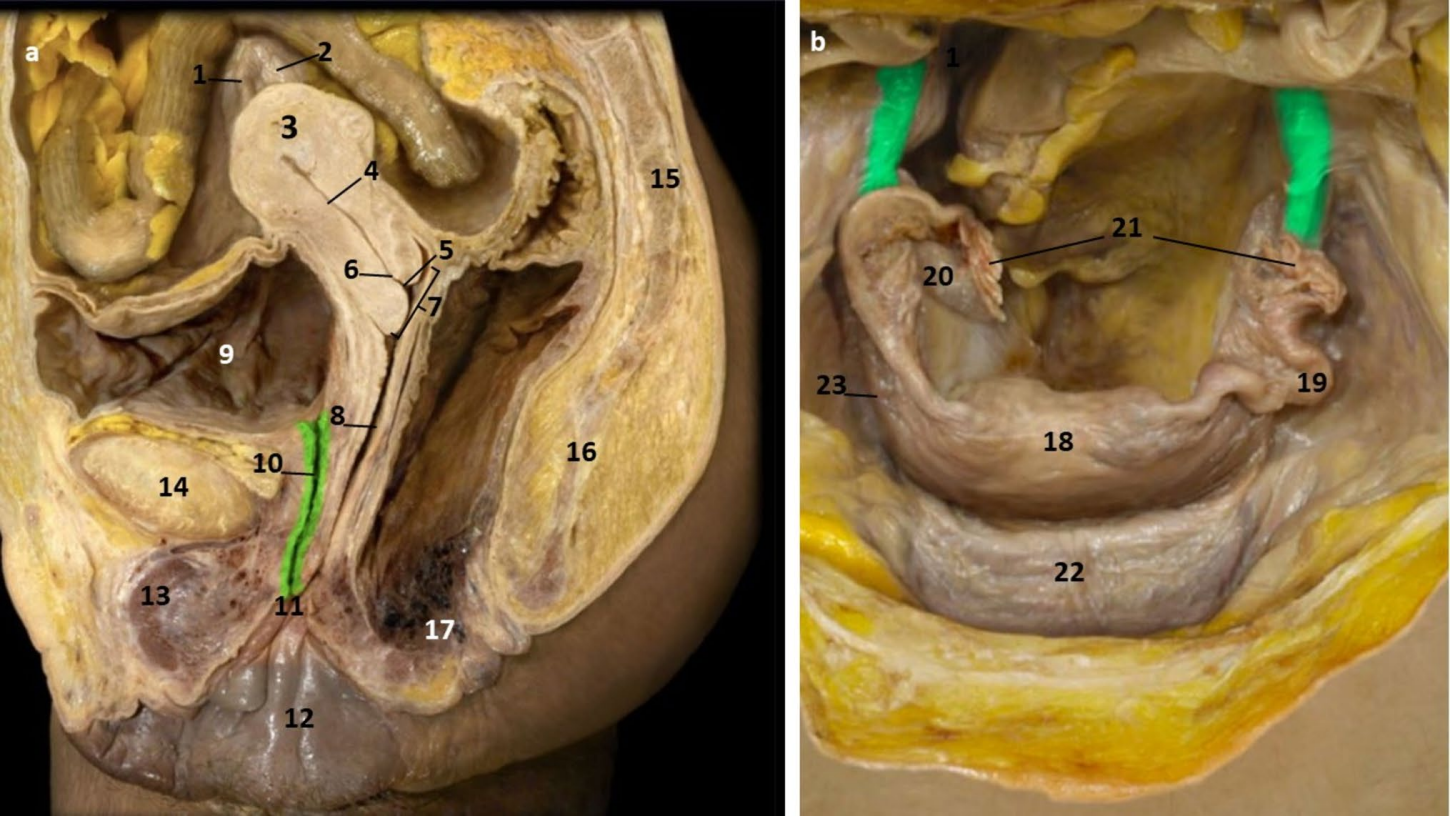
Female reproductive system. a, sagittal section; b, abdominal cavity view (curtesy of Shane McTauge, University of Iowa) **(a)** 1: Uterine tube, 2: Ovary: 3: Uterus, 4: Endometrium, 5: Cervix 6: Endocervix, 7: Ectocervix, 8: Vagina, 9: Bladder, 10: Urethra, 11: External urethral orifice, 12: Labia majora, 13: Clitoris, 14: Pubis (Symphysis pubica), 15: lumbar vertebra, 16: Coccyx, 17: Anus **(b)** 18: fundus of uterus, 19: Uterine (Fallopian tube), 20: Ovary, 21: Fimbriae of uterine tube, 22: Bladder, 23: Round ligament of uterus

In the cadaveric images (Fig. 13a and b), the female reproductive organs are shown with anatomical details and their relationships and positions within the body. Clearly visible are the uterus, cervix, Uterine (Fallopian) tubes, and ovaries. The bladder and urethra are also present. The external genitalia are shown, including the labia majora and clitoris. Leonardo’s depictions of the uterus (Figs. 11a-c) show it extended and more rotund. They are more schematic, demonstrating his speculative approach. The bladder is positioned correctly, anterior to the uterus (Fig. 11c). However, it is grossly extended - and balloon-like. The size comparison of the bladder and uterus can be seen in Fig. 13a, which may have influenced Leonardo’s exaggerated depiction. The endometrium and uterine cavity are shown in Figs. 13a and 4. Though Leonardo sketched the uterus correctly as a single cavity (Figs. 11a and b), its accuracy was not evident. Comparing Leonardo’s uterus (Fig. 11b) to the cadaveric image (Fig. 13b), we can see an attempt at anatomic accuracy. The fundus of the uterus (Fig. 13b, 18) can be recognized in his drawing. However, the ovaries and uterine tubes are misplaced or misunderstood. Moreover, Leonardo does not include the fimbriae of the uterine tubes in any of his sketches. The prominent projections emanating from the top of the uterus (Figs. 11b and 11) may represent the round ligament of the uterus, which can be seen in the cadaveric image (Fig. 13b, 23).

The structures of the female reproductive organs are easily visible in the abdominal cavity of a well-prepared, preserved, and clean cadaver (Fig. 13b). The lack of detail in Leonardo’s drawing may be due to the conditions he was dissecting and/or having fewer female cadavers available to him.

## Foetal Development

Leonardo’s fascination with embryology and foetal development is clear in his extensive notes on anatomical studies, where he expresses his intent to document the stages of a child’s development in the womb, beginning with the formation of various parts throughout the course of gestation. He writes:

*Your order shall commence with the formation of the child in the womb, saying which part of it is formed first and so on in succession, placing its parts according to the times of pregnancy until the birth, and how it is nourished, learning in part from the eggs which hens make* [5].

**Fig. 14:**
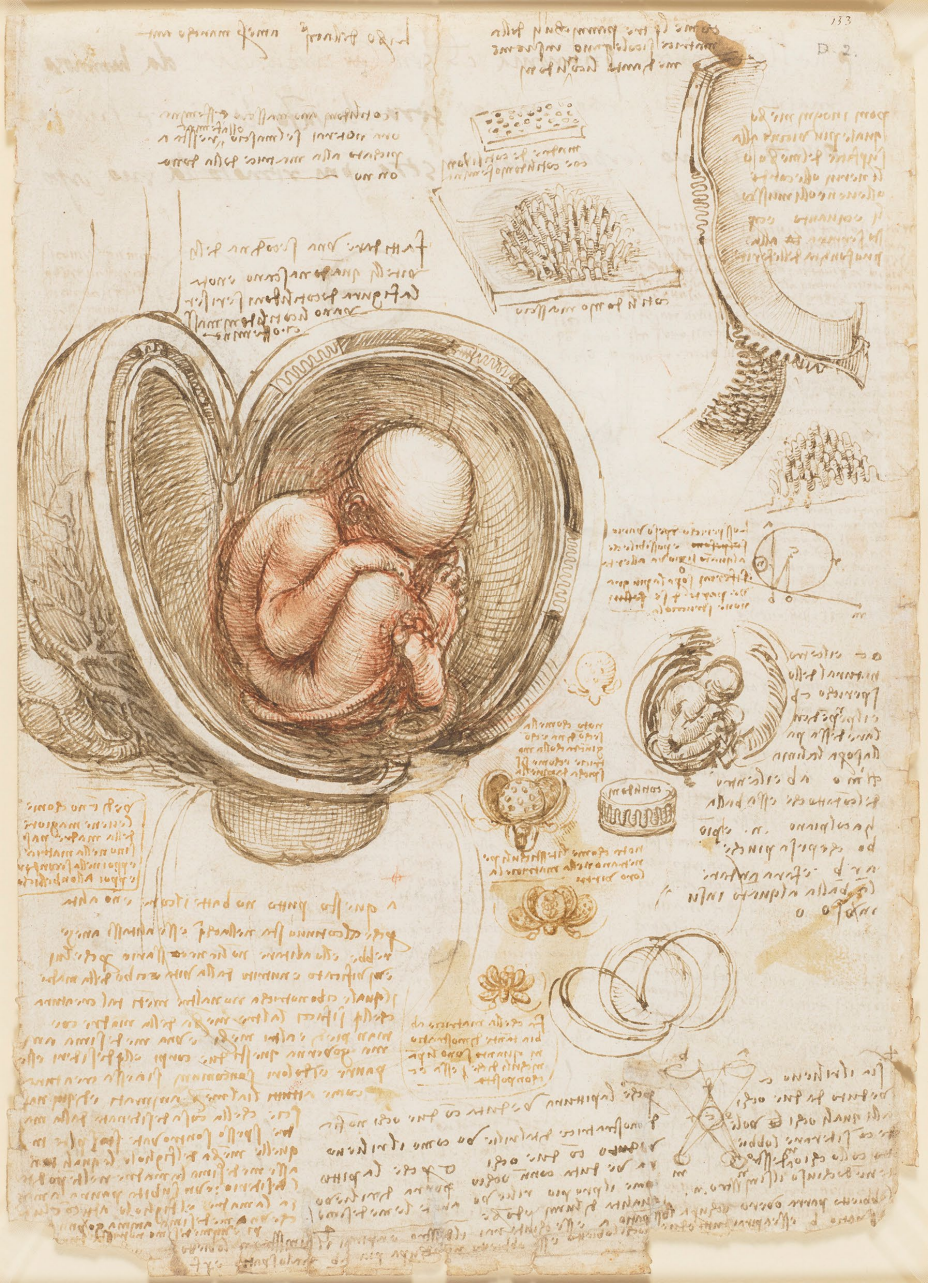
The fetus in the womb; sketches and notes on reproduction c.1511 Red chalk and traces of black chalk, pen and ink, wash. Verso: Pen and ink, with some offset red chalk, 30.4 x 22.0 cm (sheet of paper), RCIN 919102 (with permission from The Royal Collection Trust)

In Fig. 14, Leonardo depicts a foetus in utero, with the uterus exposed, alongside a smaller sketch that includes the same view and detailed representations of the placenta and uterus. The larger drawing illustrates a fetus in a complete breech position with crossed legs. The umbilical cord is wrapped around the foetus’s legs; however, its connection to the placenta is not obvious. An ovary attached to the uterus via the vas sperminarium, and associated vessels is shown on the left of the external uterus. In the geometrical diagram at the centre-right, Leonardo suggests that the weight of the foetus’s head might cause it to tip over in the uterus for a normal head-first delivery, like an eccentrically weighted sphere rolling slightly up an incline. The small sketches below the centre illustrate the uterine membranes unfurling one by one like flower petals, and in the main drawing, Leonardo uses this concept to show the membranes in cross-section [6]. Leonardo demonstrates his understanding of foetal life in utero and posits in his notes the lack of a foetal heartbeat in utero:

*In the case of this child, the heart does not beat, and it does not breathe because it lies continually in water. And if it were to breathe, it would be drowned, and breathing is not necessary to it because it receives life and is nourished from the life and food of its mother* [5].

Leonardo meticulously illustrates the uterus with its blood vessels, along with the foetal membranes and the umbilical cord, complete with its vessels. However, he mistakenly depicted the placental attachment as cotyledonary, resembling that of bovine with the foetal cotyledons interlocking with the maternal crypts [10]. He writes in his notes:

*Just as the fingers of the hand are interwoven, one in the interval of the other... so the fleshy villi of these little sponges [cotyledons] are interwoven like burrs, one half with the other. He also observed, the vessels of the infant do not branch into the substance of the uterus of its mother, but into the secundines [placenta], which act like a shirt lining the interior of the uterus, to which it is connected (but not united) by means of the cotyledons* [21].

His drawings and conclusions were influenced by extrapolations from bovine (and other mammal) dissections. He extensively details the bicornuate uterus of a gravid cow [6, 21].

Leonardo’s depiction of the spherical uterus may also have been a product of the religious dogma of the time. Following this dogma, it has been suggested that the uterus was considered the spiritual centre of womanhood, and thus it was imagined as a flawless sphere [23].

Leonardo carried out a dissection of a 4-month-old foetus, thus enabling him to make some observations on fetal growth. Through this dissection, Leonardo corrected earlier errors regarding the umbilical vessels, clarifying that the umbilical vein extends to the liver and serves dual functions—carrying nutrients and urine. He made significant observations on foetal growth, muscle mechanics, including the role of the biceps brachii and brachialis muscles in arm movement. Leonardo also speculated on foetal nutrition, believing menstrual blood nourished the foetus through the umbilical vein. His work combined detailed anatomical sketches with hypotheses on foetal development and function [21].

To further his understanding of foetal development, he had intentions of studying chick development. He writes in his notes:

*But you should make first the anatomy of the hatched egg…. see how birds are nourished in their eggs* [5].

However, more detailed discoveries were made in 1672 when Marcello Malpighi published the detailed microscopic account of chick development [24].

Leonardo’s work in embryology, though limited by the knowledge of his time, demonstrated his deep curiosity and innovative approach to understanding foetal development. His detailed observations, blending art and science, laid a foundation for future anatomical studies. As with a lot of his anatomy investigations, some of his conclusions were influenced by animal comparisons.

## Discussion

Leonardo da Vinci is widely celebrated as one of the greatest artists in history, known for iconic works like The Last Supper and Mona Lisa, which highlight his exceptional skill in portraying the human form and emotion. His reputation as an artist is equally supported by his deep curiosity and inventive nature, which extended well beyond painting.

From 1508 to 1513, he primarily devoted himself to scientific pursuits, only occasionally returning to paintings he had begun earlier. Among his various scientific interests—which included optics, geology, botany, and hydrodynamics [2]. The field that captivated him the most and where he made his most significant discoveries was human anatomy [6, 7]. Furthermore, Leonardo’s intention to compile a treatise on anatomy is reflected in his notes c1489:

*This work should commence with the conception of man, and should describe the nature of the womb, and how the child inhabits it, and in what stage it dwells there, and the manner of its quickening and feeding, and its growth, and what interval there is between one stage of growth and another, and what thing drives it forth from the body of the mother, and for what reason it sometimes emerges from the belly of its mother before the due time. Then you should describe which are the limbs that grow more than the others after the child is born; and give the measurements of a child of one year* [5].

It was through his collaborations with Marcantonio della Torre that this book would be completed. Unfortunately, Marcantonio died in 1511 of the plague. This ended their collaboration and thus ceased their anatomical studies. As a result, the anticipated anatomy book was never completed. The loss of this work was a profound setback, depriving valuable insights and knowledge that could have advanced the understanding of human anatomy – preceding the work of Andreas Vesalius [2].

Leonardo’s anatomical research ended after his move to France in 1516, and there is no indication that he ever tried to organise his research for publication. Upon his death in 1519, he left his papers to his assistant, Francesco Melzi. Although Leonardo’s anatomical studies were mentioned by his early biographer Vasari, their dense and disorganised nature made them difficult to comprehend [2].

Because they were never published, these studies were essentially lost to the world. Meanwhile, anatomical research progressed elsewhere, culminating in Andreas Vesalius’s groundbreaking work, *De humani corporis fabrica* (On the Fabric of the Human Body), published in 1543 [25]. The 150 surviving sheets of Leonardo’s anatomical studies eventually made their way to England in the seventeenth century, where they were incorporated into the Royal Collection, bound together with 450 of his other artistic drawings. However, it was not until 1900 that these studies were finally published and understood. By then, their potential influence on the development of anatomical knowledge had long diminished. While Leonardo’s paintings revolutionised European art, his anatomical investigations—some of the most advanced of their time—remained largely unknown [6].

Leonardo’s notes and sketches were published between 1898 and 1916 as facsimile editions, and then in a conservation effort, in the early 1970s, all his drawings, many with related notes, were arranged together and then published in 1979 [6].

Leonardo da Vinci is now recognised as both a great anatomist and one of the most accomplished artists and draughtsmen in history [8]. His depictions of several anatomical structures and functions have been corroborated through modern-day medical research [26, 27]. These include the brain, peripheral nervous system, and neuroanatomy [28–31]; the heart, aortic valves, and circulation [32–34, 11]; and the position of the fetus in utero [35]. He even postulated the laws of inheritance in his notes:

*The black races in Ethiopia are not the product of the sun; for if black gets black with child in Scythia, the offspring is black; but if a black gets a white woman with child the offspring is grey. And this shows that the seed of the mother has power in the embryo equally with that of the father* [5].

However, despite his meticulous dissections and detailed drawings - much of his interpretation was erroneous. His anatomical investigations, and his depictions and errors regarding the musculoskeletal system, cardiovascular system, nervous system, and other organs were, in part, influenced by his Galenic beliefs and the practical limitations of the time [9]. Leonardo performed dissections on animals, documenting these studies in his notebooks [10]. Like Galen, who only dissected animals and applied those findings to human anatomy, Leonardo might have adopted a similar approach making assumptions where he found ‘anatomical gaps’ in his human dissections [9]. For instance, the absence of a prostate gland in all his drawings of the male reproductive system is obvious, and the reason for this omission may be because his anatomical investigations were conducted in castrated oxen with atrophied prostate glands [36].

His early anatomical sketches depicting humans in coitus (Figs. 2 and 3) were not derived from observations of human dissections, but more likely based on a blend of the predominant Galenic notions of the time, animal dissection, and speculation [37]. However, another suggestion is that these early drawings are demonstrating Leonardo’s thinking of an overall heuristic model of physiological links of the human body [38], where Leonardo is working to understand the human body as part of a larger system with his anatomical investigations more aligned with philosophical inquiry than with the clinical practice of medicine [25].

Leonardo believed that all mammals shared similar reproductive structures, and in his embryological studies conducted a few years later, he applied the cotyledonous placental structure he had observed in cows to the human form [6]. His fusion of animal dissections with this human anatomical observation can be seen in his rendering of the female reproductive anatomy (Fig. 12), where he draws a large spherical uterus more akin to that of a cow than a human [6]. However, his depiction of the human uterus improved in later sketches (Fig. 11b), where a single uterine cavity is drawn, with more appropriate uterine proportions and cervix [21], and he did correctly describe that the foetal vasculature is not continuous with that of the mother [39].

His studies on foetal development were also influenced by animal dissections – in particular bovine. However, he did make some interesting studies on a human foetal dissection – where he was able to make more detailed studies on the umbilical cord, but still made some conclusions on how the foetus is nourished in utero based on Galenic notions [21].

Over time, Leonardo developed into a highly skilled anatomist, performing delicate, intricate dissections, and creating detailed sketches of his observations. The direct influence of Galen’s teachings was not always apparent in his work, and the anatomical inaccuracies present were more likely due to the practical challenges of his era [9].

One major challenge when studying human anatomy was the lack of any preservation procedures to keep cadavers from putrefying. When a cadaver was made available, time became a main issue. It was therefore common practice to dissect the abdominal cavity first, as it contained organs that putrefied most easily, followed by the thorax, head, and extremities. To prevent putrefaction, dissections were scheduled in winter when the weather conditions were more suitable to preserve the organs at best [4].

Even though his dissections were conducted mostly during the winter, the lower temperatures were not always sufficient to delay putrefaction, which would have affected the quality of the specimens available to him for anatomical studies. He writes of his experience in his notes:

*But though possessed of an interest in the subject, you may perhaps be deterred by natural repugnance, or if this does not restrain you then perhaps by the fear of passing the night hours in the company of these corpses quartered and flayed, and horrible to behold, and if this does not deter you then perhaps you may lack the skill in drawing essential for such representation* [5].

Leonardo created highly detailed illustrations and studies of various anatomical systems, with his work on the musculoskeletal and nervous systems standing out as particularly notable. He meticulously illustrated nearly every bone in the body, along with many major muscle groups. Although he occasionally included nerves and blood vessels, his primary focus was on the biomechanics of the body [6]. His detailed images are comparable to the standards of today’s anatomical texts. However, his depiction of reproductive anatomy lacks the same level of detail.

Several factors might explain this:

(i) The science of reproduction was not well understood at the time and was heavily influenced by Hippocratic-Galenic concepts, making functional induction less straightforward than those of the musculoskeletal system or the heart.
(ii) The reproductive organs may have been difficult to dissect from the abdomen under the conditions of the Florentine climate. This might explain why Leonardo incorporated elements of animal reproductive anatomy into his studies of humans to construct a more comprehensive understanding of physiology.
(iii) It may be possible that he simply got distracted and focused on other matters that interested him more.

He was notorious for not completing commissioned works of art to the frustration of his many patrons [40]. It is feasible that Leonardo’s lack of real detail in his rendering of the reproductive system (when compared to his other anatomical studies) was due to his restlessness and constant search for novelty and learning. It has been suggested that Leonardo may have had Attention-Deficit/Hyperactivity Disorder (ADHD), a neurodevelopmental disorder characterized by persistent patterns of inattention, hyperactivity, and impulsivity that interfere with functioning or development [41]. Leonardo’s notes demonstrate a level of disorganization seen with ADHD, with many unrelated writings filling the pages (though, this may be due to reuse of pages at different times). However, ADHD can offer certain advantages: mind wandering may inspire creativity and originality, while restlessness can drive the pursuit of novelty and a desire for change – and Leonardo certainly demonstrated that through his sketches and notes.

It is unfortunate that Leonardo left no publications during his lifetime. His notes, consisting of over 6000 pages, of which 190 deal with anatomy, were extensive and disordered. And these are the ones we are aware of. It is suggested that as much as three quarters to four fifths is missing [42]. We will likely never fully know the extent of his anatomical discoveries or the insightful functional deductions he made through his investigations. What is known is that under proper mentorship and guidance, his works could have been organized into several volumes, making his material accessible to scholars in the centuries that followed.

Despite his distractions and procrastinations, the prevailing – incorrect - scientific and philosophical concepts, and the inadequacy of the tools and instruments available to him in this period, Leonardo’s accomplishments evoke the idea of a genius. A true polymath whose investigations in many areas covering astronomy, engineering, botany, geology, and anatomy ensure his legacy endures. His notes describe his approach as scientific, with keen observation and experimentation - he could be considered the first scientist [1].

## Supplementary Information

Below is the link to the supplementary material.

ESM 1 (DOCX 342 KB)

## Data Availability

Not applicable.
