# Supplementary figures and images for: The Renaissance of Reproductive Science: Leonardo da Vinci’s Anatomical Contributions
Source: Reprod Sci. 2025 Jan 16;32(3):575–99. doi: 10.1007/s43032-024-01772-9 (PMC11870949; doi:10.1007/s43032-024-01772-9)

**
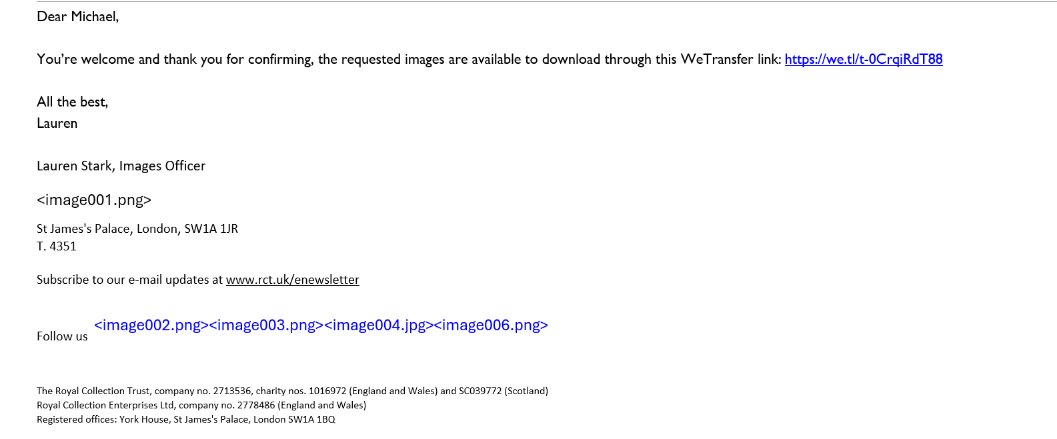

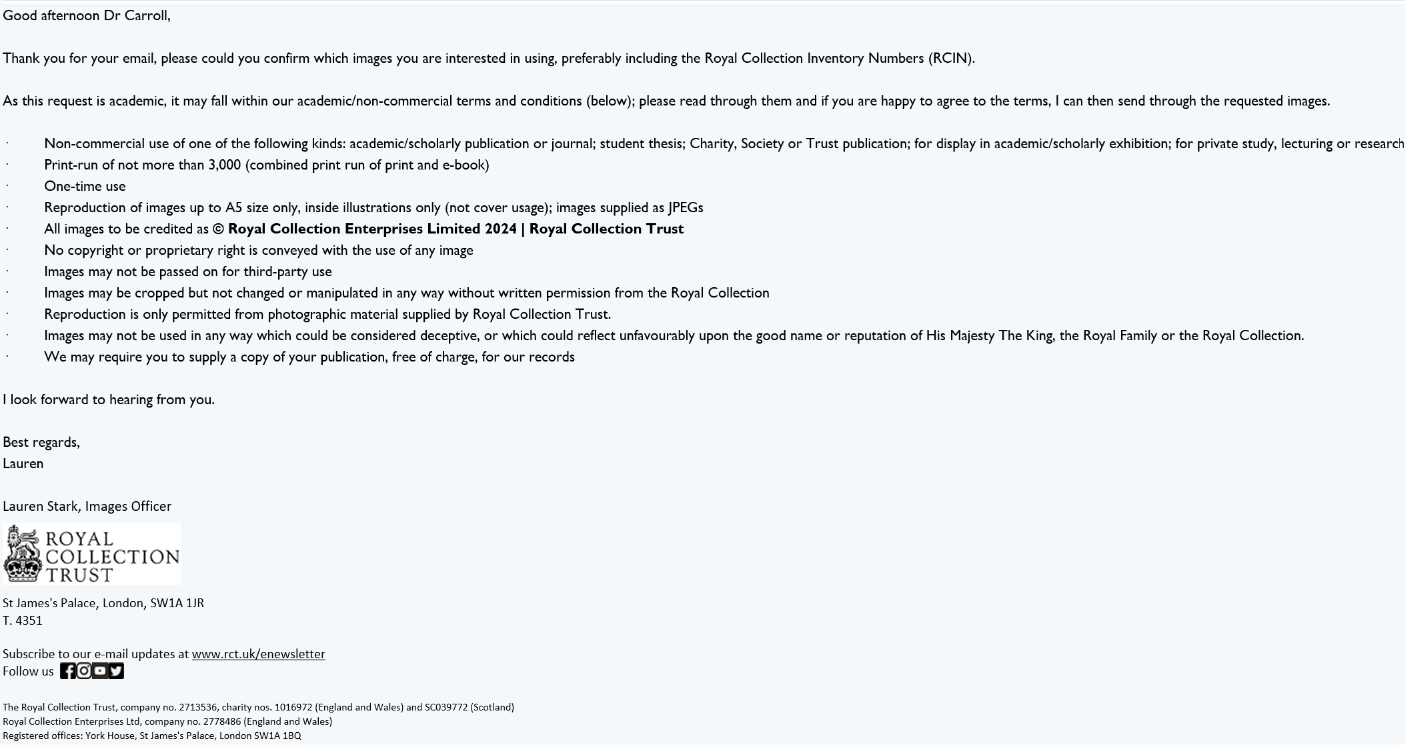

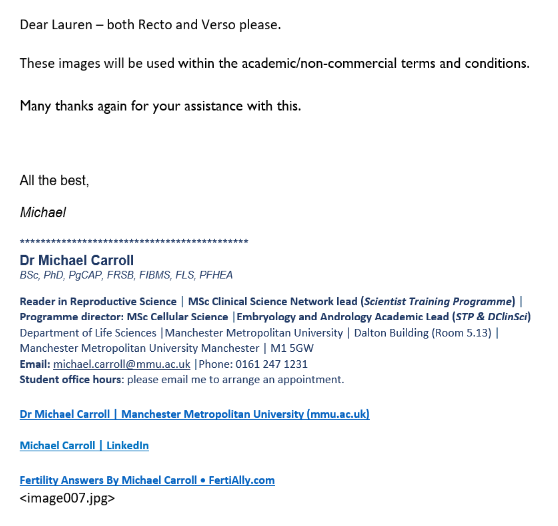
Royal Collection Trust Permission**

Supplement: Supplementary file 1 — (DOCX 342 KB) [file 43032_2024_1772_MOESM1_ESM.docx]
